# The tumor suppressor LACTB remodels mitochondria to promote cytochrome c release and apoptosis

**DOI:** 10.1126/sciadv.adx7809

**Published:** 2025-11-12

**Authors:** Sukrut C. Kamerkar, Taewook Kang, Radu V. Stan, Edward J. Usherwood, Henry N. Higgs

**Affiliations:** ^1^ Department of Biochemistry and Cell Biology, Geisel School of Medicine at Dartmouth, Hanover, NH 03755, USA.; ^2^ Department of Microbiology and Immunology, Geisel School of Medicine at Dartmouth, Hanover, NH 03755, USA.

## Abstract

Mitochondria are pivotal regulators of cellular homeostasis, integrating energy metabolism, biosynthesis, and programmed cell death (apoptosis). During apoptosis, mitochondrial outer membrane permeabilization by BCL-2-associated X protein/BCL-2 Homolog Antagonist Killer (BAX/BAK) pores facilitates release of apoptotic factors, while the role of inner mitochondrial membrane (IMM) remodeling remains less understood. Here, we identify serine beta-lactamase-like protein (LACTB), a filament-forming serine protease and tumor suppressor, as a regulator of IMM dynamics during apoptosis. LACTB suppression reduces cytochrome c release and apoptosis, whereas its overexpression promotes these effects. LACTB does not affect BAX or Drp1 recruitment to mitochondria. Rather, LACTB is required for apoptosis-induced mitochondrial remodeling, independent of OPA1 processing. Intriguingly, LACTB knockdown does not affect mitochondrial shape changes induced by CCCP treatment, suggesting that LACTB action is apoptosis-specific. Purified LACTB binds and remodels cardiolipin-enriched membrane nanotubes preferentially over planar lipid membranes, suggesting a direct effect in apoptotic membrane remodeling. Collectively, our findings suggest LACTB to be a mediator of apoptosis-induced IMM remodeling, a possible mechanism for tumor suppression in cancer.

## Teaser

LACTB remodels the inner mitochondrial membrane during apoptosis to facilitate the release of cytochrome c and other factors.

## INTRODUCTION

Mitochondria are indispensable regulators of cellular life and death (*1*). Beyond their well-established role in adenosine 5′-triphosphate (ATP) production via oxidative phosphorylation, mitochondria are hubs for the biosynthesis and metabolism of signaling molecules, lipids, and metabolites critical to maintain cellular homeostasis as well as inflammation and immune responses (*2*, *3*). Paradoxically, mitochondria are equally central to programmed cell death (apoptosis), where mitochondrial outer membrane permeabilization (MOMP) mediated by BCL-2 (B-cell lymphoma 2) family proteins (notably, BAX and BAK) leads to the release of factors that activate the apoptotic cascade, including caspase-activating proteins (cytochrome c), proteins inhibiting caspase inhibitors (SMAC/DIABLO and HTRA2/Omi), and proteins participating in DNA cleavage [apoptosis-inducing factor (AIF) and endonuclease G (EndoG)] (*4*–*7*).

While notable progress has been made in understanding the mechanisms driving MOMP, less is known about the molecular players orchestrating inner mitochondrial membrane (IMM) remodeling during apoptosis (*8*). Apoptotic IMM remodeling might be important for the release of cytochrome c, ~85% of which is sequestered in cristae where it is electrostatically bound to the IMM (*8*, *9*). Several mechanisms for apoptotic IMM remodeling have been proposed, including the actions of proapoptotic BH3-only proteins (*9*, *10*), effects of BAX/BAK beyond MOMP, and regulation of proteolytic processing of the IMM dynamin optic atrophy 1 (OPA1) (*8*, *11*, *12*).

LACTB is a filament-forming serine protease localized to the mitochondrial intermembrane space (IMS) (*13*). Although its physiological role in noncancerous cells remains unclear, LACTB is well established as a tumor suppressor in several cancer types (*14*–*22*). However, the mechanism underlying this tumor-suppressive activity is still under debate. One study suggests that LACTB mediates the proteolysis of mitochondrially localized phosphatidylserine decarboxylase (PISD), thereby altering mitochondrial phospholipid synthesis (*14*). Other reports have implicated cytoplasmic functions for LACTB in tumor suppression through interactions with p53, protein phosphatase 1α (PP1A), or components of the phosphatidylinositol 3-kinase (PI3K) pathway (*15*, *16*, *20*). In addition, LACTB overexpression (OE) has been shown to increase apoptosis (*17*), although the mechanism remains elusive. Here, we demonstrate a direct role for LACTB in promoting the release of mitochondrial proapoptotic proteins via remodeling of the IMM, thereby establishing LACTB as a novel apoptotic factor.

## RESULTS

### LACTB plays a role in apoptosis

**Fig. 1.**
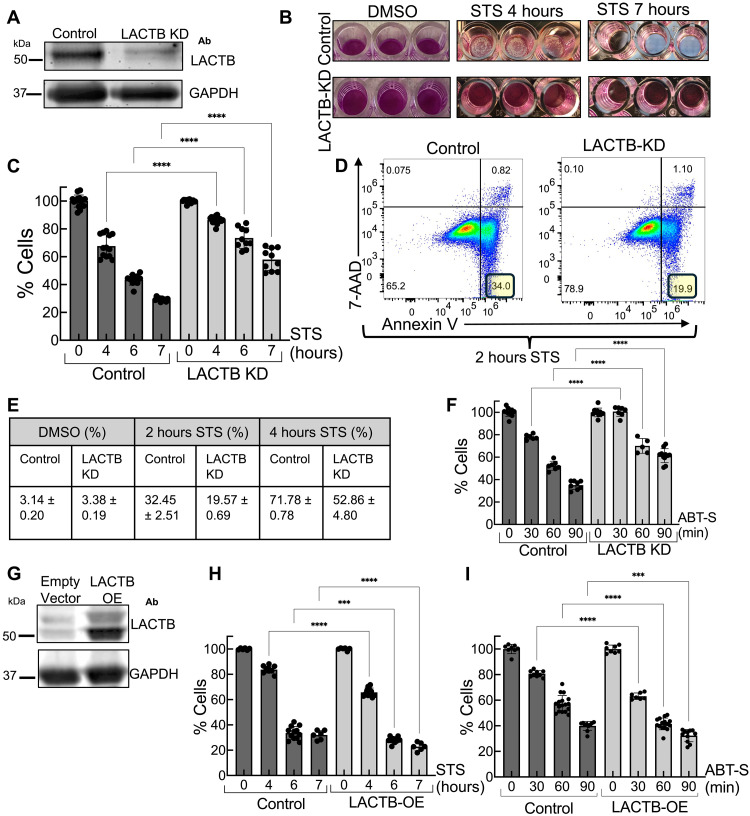
LACTB plays a role in apoptosis. (**A**) Western blot analysis of LACTB in LACTB KD and control HeLa cells. Glyceraldehyde-3-phosphate dehydrogenase serves as a loading control. (**B**) SRB assay measuring cell viability in control and LACTB KD HeLa cells treated with DMSO or 1 μM staurosporine (STS) for 4 and 7 hours. (**C**) Quantification of SRB assay in control and LACTB KD cells following STS treatment (*N* = 3). (**D**) Flow cytometry analysis of annexin V and 7-AAD staining in control and LACTB KD cells treated with 1 μM STS for 2 hours. (**E**) Table quantifying the percentage of annexin V^+^/7-AAD^−^ cells after treatment with STS (2 and 4 hours) or DMSO (4 hours) (*N* = 3). (**F**) SRB assay of control and LACTB KD cells upon treatment with ABT-737 (10 μM) and S63845 (2 μM) (ABT-S) (*N* = 2). (**G**) Western blot analysis of LACTB in control and stable LACTB OE HeLa cells. (**H**) SRB assay of control and LACTB OE cells following 1 μM STS treatment (*N* = 2). (**I**) SRB assay of control and LACTB OE cells following ABT-S treatment (*N* = 2). One-way analysis of variance (ANOVA), ****P < 0.0001; ***P < 0.001. Data are presented as means ± SD. *N* indicates the number of independent experiments. SRB assay measures relative protein content. Ab, antibody.

One proposed mechanism for LACTB-mediated tumor suppression is its involvement in apoptosis (*17*). To test this, we performed LACTB knockdown (KD) in HeLa cells. The KD was highly efficient: Immunofluorescence staining showed loss of mitochondrial LACTB signal in 98.5 ± 1.7% of cells (fig. S1, A to C), and Western blot analysis of whole-cell extracts confirmed a comparable reduction in LACTB levels (Fig. 1A). We then induced apoptosis using staurosporine (*23*) in control and LACTB KD HeLa cells and assessed cell viability using the sulforhodamine B (SRB) assay (*24*).

LACTB KD cells display increased cell viability compared to controls at 4, 6, and 7 hours of staurosporine treatment (Fig. 1, B and C). LACTB KD causes a similar apoptotic decrease in B16-F10 cells (fig. S1, D and E). As an independent method to assess apoptosis, we stained control and LACTB KD cells with annexin V and 7-AAD (*25*). LACTB KD cells display reduced apoptotic cell percentage compared to control upon staurosporine treatment (Fig. 1, D and E, and fig. S1, G to L). As an alternative apoptosis inducer, we treated cells with a combination of ABT-737 (Bcl-2 inhibitor) (*26*) and S63845 [myeloid cell leukemia 1 (MCL-1) inhibitor], and abbreviate this combination as ABT-S, (*27*) which activates the BAX pore (*28*, *29*). As with staurosporine, LACTB KD cells display increased cell viability upon this treatment (Fig. 1F and fig. S1F).

Previous studies on LACTB tumor suppression showed that LACTB OE caused a decrease in cell proliferation and tumor growth (*14*, *15*, *17*–*19*). We therefore stably overexpressed LACTB in HeLa cells (Fig. 1G) and tested their response to apoptosis. LACTB OE cells display increased sensitivity to cell death induced by staurosporine (Fig. 1H) or ABT-S (Fig. 1I). Together, these results suggest that LACTB plays a role in apoptosis, with LACTB KD delaying apoptosis and LACTB OE accelerating apoptosis.

### LACTB is involved in release of mitochondrial proteins during apoptosis

Next, we investigated the mechanism by which LACTB enhances apoptosis. A previous study (*14*) suggested that LACTB functions by proteolytically degrading the PISD enzyme. However, we did not detect significant changes in PISD levels upon LACTB KD or OE (fig. S2, A and B), similar to findings by others (*15*, *16*, *30*). We therefore sought alternate mechanisms.

**Fig. 2.**
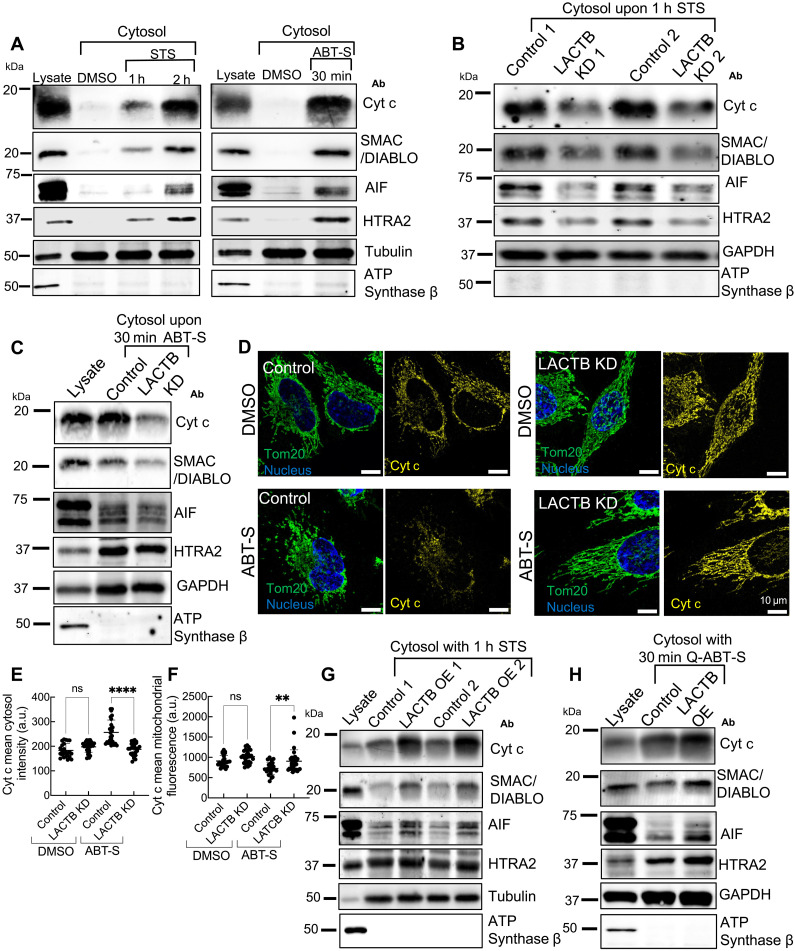
LACTB enhances mitochondrial release of cytochrome c and other factors upon apoptosis. (**A**) Western blot showing time-course analysis of mitochondrial apoptotic factor release into the cytosol following 1 μM STS or ABT-S treatment in HeLa cells. A total of 6 and 10 μg of protein were loaded per sample for STS and ABT-S treatment, respectively. (**B**) Western blot showing mitochondrial apoptotic factor release into the cytosol after 1 μM STS treatment for 1 hour in control or LACTB KD HeLa cells. A total of 14 μg of protein was loaded per sample. (**C**) Western blot showing mitochondrial apoptotic factor release into the cytosol after ABT-S treatment for 30 min in control or LACTB KD HeLa cells. A total of 12 μg of protein was loaded per sample. (**D**) Fluorescence staining of cytochrome c (Cyt c), Tom20, and DNA [4′,6-diamidino-2-phenylindole (DAPI)] in control and LACTB KD HeLa cells under DMSO and ABT-S treatment for 30 min. (**E** and **F**) Quantification of cytosolic cytochrome c levels (E) and mitochondrial cytochrome c retention (F) from fluorescence staining experiments shown in (D). *N* ≥ 23 cells, One-way ANOVA, ****P < 0.0001; **P < 0.01. (**G**) Western blot showing mitochondrial apoptotic factor release into the cytosol after 1 μM STS treatment for 1 hour in control or LACTB OE HeLa cells. A total of 10 μg of protein was loaded per sample. (**H**) Western blot showing mitochondrial apoptotic factor release into the cytosol after ABT-S treatment for 30 min in control or LACTB OE HeLa cells. Cells treated with the pan-caspase inhibitor Q-VD-OPh (20 μM, 1-hour preincubation) prior to ABT-S treatment. A total of 12 μg of protein was loaded per sample. a.u., arbitrary unit; h, hour; ns, not significant.

MOMP is often a committed step in apoptosis, leading to the release of mitochondrial proteins such as cytochrome c, SMAC/Diablo, AIF, and HTRA2/Omi into the cytoplasm (*1*, *31*–*33*). Using differential centrifugation to separate cytoplasm from mitochondria (*31*), we observe an increase in cytoplasmic levels of these mitochondrial proteins upon treatment with staurosporine or ABT-S in control cells (Fig. 2A). LACTB KD causes a significant reduction in cytosolic release of these mitochondrial proteins induced by either stimulus (Fig. 2, B and C). The overall levels of these proteins remain unchanged with either LACTB KD or OE (fig. S2, C and D).

In addition, we used immunofluorescence to monitor cytochrome c release. In control HeLa cells, cytochrome c staining is concentrated in mitochondria, but a significant portion translocates to the cytosol after 30 min of ABT-S treatment (Fig. 2D). LACTB KD reduces cytochrome c translocation (Fig. 2, D to F). Similar results occur in U2-OS cells with staurosporine treatment (fig. S2, E to G).

We next tested whether LACTB KD also influences downstream apoptotic events, such as caspase-3 activation and poly(ADP-ribose) polymerase (PARP) cleavage. Western blot analysis reveals a delay in PARP cleavage following staurosporine treatment in LACTB KD cells (fig. S3A). In addition, the 30-kDa full-length form of caspase-3 decreases at a slower rate in LACTB KD cells than in control cells upon staurosporine treatment (fig. S3B), suggesting delayed caspase-3 activation.

Last, we tested the effect of LACTB OE on mitochondrial release of cytochrome c and other factors. LACTB OE leads to increased staurosporine-induced release of mitochondrial factors (Fig. 2G). To determine whether this effect is independent of caspase activity, we incubated cells with the pan-caspase inhibitor Q-VD-OPh prior to apoptosis induction (*34*). Even under these conditions, LACTB OE increases the Q-ABT-S–induced release of these mitochondrial proteins (Fig. 2H). In subsequent experiments, we use caspase inhibition to prevent cell detachment while imaging.

Together, these results suggest that LACTB is required for efficient release of mitochondrial proteins during apoptosis, as well as for downstream events such as caspase-3 activation and PARP cleavage. The observation that LACTB KD delays, but does not block, these steps supports a facilitative role for LACTB in apoptotic progression.

### LACTB is required for mitochondrial remodeling upon apoptosis

**Fig. 3.**
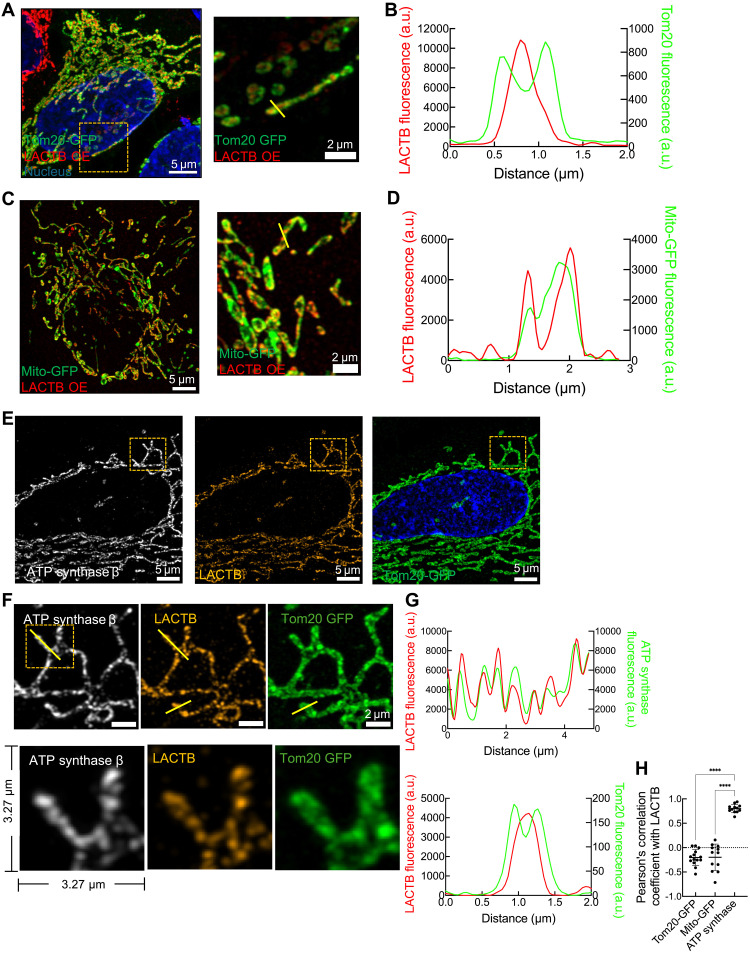
LACTB colocalizes with ATP synthase in mitochondria. (**A**) Immunofluorescence staining of LACTB (red) in LACTB OE HeLa cells transiently transfected with Tom20-GFP (green). DAPI, blue. Image on the right shows zoom of the boxed region. (**B**) Line profile through a mitochondrion in (A). (**C**) Immunofluorescence staining of LACTB (red) in LACTB OE HeLa cells transiently transfected with Mito-GFP (green). DAPI, blue. Image on the right shows zoom of the boxed region. (**D**) Line profile through a mitochondrion in (C). (**E**) Immunofluorescence staining LACTB (orange) and ATP synthase β (gray) in LACTB OE HeLa cells transiently expressing Tom20-GFP (green). DAPI, blue. (**F**) Zoomed image from box in (E), comparing localization pattern of ATP synthase β, LACTB, and Tom20. Images below show a further zoom of the mitochondrion in the line profile analysis in (G). (**G**) Line profile analysis of LACTB-ATP synthase β and LACTB-Tom20 signals from (F). (**H**) Pearson’s colocalization coefficient for colocalization between LACTB and Tom20-GFP, Mito-GFP, or ATP synthase. One-way ANOVA, ****P < 0.0001. Data are presented as means ± SD. *N*_mito_ ≥ 12.

Next, we asked how LACTB might participate in release of mitochondrial factors during apoptosis. We first tested the findings of an earlier study (*13*) that suggested LACTB to be present in the mitochondrial IMS. We reexamined LACTB localization by immunofluorescence using Airyscan microscopy in HeLa cells stably overexpressing untagged LACTB, relative to an outer mitochondrial membrane marker [Tom20–green fluorescent protein (GFP)] and a mitochondrial matrix marker (Mito-GFP). LACTB staining is consistently found within the Tom20 signal (Fig. 3, A and B) and surrounding the Mito-GFP signal (Fig. 3, C and D). We also performed immunofluorescence staining for LACTB and ATP synthase β subunit (on the matrix side of cristae). The LACTB and ATP synthase signals display strong colocalization [Fig. 3, E, F (magnified insert), and G (line profile)], as supported by a positive correlation analysis (Fig. 3H). These results suggest that LACTB localizes to the IMM and possibly enriches in cristae.

One possible mechanism for LACTB-mediated enhancement of mitochondrial protein release is through facilitating BAX/BAK channel assembly on the outer mitochondrial membrane (*35*). First, we checked BAX and BAK levels in control, LACTB KD, and LACTB OE cells and observed no significant differences (fig. S4A). BAK is single-pass transmembrane protein constitutively present on the outer mitochondrial membrane (OMM), while BAX is recruited to the OMM from the cytosol upon apoptotic stimulation (*36*). To assess whether LACTB KD affects mitochondrial BAX recruitment, we immunostained for BAX under apoptotic and nonapoptotic conditions. Both control and LACTB KD cells display negligible mitochondrial BAX under dimethyl sulfoxide (DMSO) treatment (fig. S4B). Upon Q-ABT-S treatment, BAX punctae accumulate on the mitochondria in both control and LACTB KD cells (fig. S4, C and D). Cell fractionation analysis further corroborates these findings (fig. S4E). We also find that BAX-positive LACTB KD cells retain cytochrome c staining, in contrast to control cells, which display a marked loss of cytochrome c signal (fig. S5, A and B). These results suggest that LACTB facilitates mitochondrial protein release without directly affecting BAX recruitment to mitochondria.

**Fig. 4.**
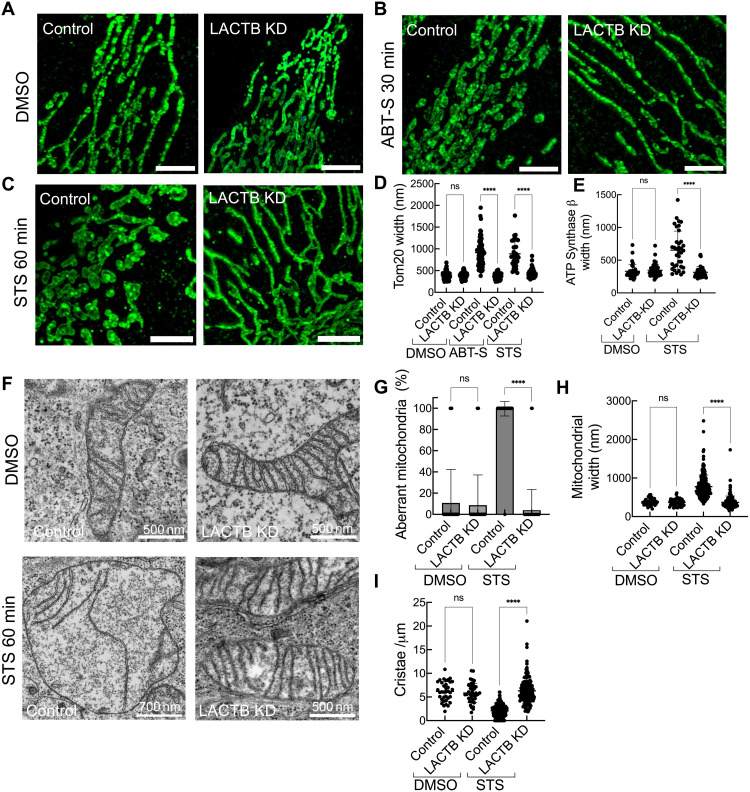
LACTB mediates mitochondrial remodeling upon apoptosis induction. (**A** to **C**) Immunofluorescence staining of Tom20 (green) in control siRNA and LACTB KD HeLa cells treated with DMSO [(A); 1 hour], ABT-S [(B); 30 min], or STS [(C); 1 hour]. Scale bars, 5 μm. (**D** and **E**) Quantification of mitochondrial width changes under DMSO, ABT-S, or STS treatment [as in (A) to (C)] using Tom20 staining (D) or ATP synthase β staining (E). Widths determined by Gaussian fitting and full-width at half-maximum analysis. *N* = 2 independent experiments. *N*_mito_ (STS) ≥ 40; *N*_mito_ (ABT-S or DMSO) ≥ 75. (**F**) Transmission electron microscopy (TEM) micrographs of mitochondria in control and LACTB KD cells under DMSO or STS treatment for 1 hour. (**G**) Quantification of aberrant mitochondria from analysis of TEM micrographs of control and LACTB KD HeLa cells under DMSO or STS treatment for 1 hour. (**H**) Mitochondrial widths measured from TEM micrographs of control and LACTB KD HeLa cells under DMSO or STS treatment for 1 hour. (**I**) Quantification of cristae density (number per micrometer) from TEM micrographs of control and LACTB KD HeLa cells under DMSO or STS treatment for 1 hour. For [(F) to (I)], all quantifications were performed in a blinded manner. Error bars represent means ± SD. Control DMSO_mito_ = 38; LACTB KD DMSO_mito_ = 46; control STS_mito_ = 213; LACTB KD STS_mito_ = 153. *N* = 3 independent experiments. One-way ANOVA, ****P < 0.0001.

Another possibility is that LACTB mediates changes within mitochondria that facilitate mitochondrial protein release. The IMM has been reported to undergo extensive remodeling during apoptosis (*9*, *37*). We examined mitochondrial morphology in control and LACTB KD HeLa cells, using Tom20 immunostaining and Airyscan microscopy. In the absence of apoptotic stimulus (DMSO control), LACTB KD results in slightly elongated mitochondria (fig. S6A). Upon treatment with staurosporine or Q-ABT-S, control mitochondria change morphology substantially (Fig. 4, A to C, and fig. S6, B and C), with one change being an increase in mitochondrial width (fig. S6D). In contrast, mitochondria in LACTB KD cells do not change noticeably in morphology (Fig. 4, B and C, and fig. S6, B to D). Quantification of mitochondrial width shows that control and LACTB KD cells display statistically indistinguishable mitochondrial widths in the absence of apoptotic stimulation (Fig. 4D). Upon staurosporine or Q-ABT-S treatment, mitochondria in control cells display an approximate twofold increase in width, while those in LACTB KD cells retain widths similar to DMSO treatment (Fig. 4D). We further tested these results by using ATP synthase as a marker for mitochondrial width. While the overall widths measured for ATP synthases are ~100 nm smaller than for Tom20 (which is expected, due to their relative locations), a similar trend occurs, with LACTB KD eliminating the width change induced by apoptotic stimulation (Fig. 4E and fig. S6, E to H).

To examine ultrastructural changes during apoptosis, we performed thin-section electron microscopy (EM) on control and LACTB KD cells. Consistent with the fluorescence microscopy results, mitochondrial widths are unchanged by LACTB KD under DMSO treatment. Upon staurosporine treatment, control cells display extensive mitochondrial swelling and large-scale morphological changes, whereas LACTB KD cells do not (Fig. 4, F and G, and fig. S7, A and B). Quantification of thin-section EM images reveals an approximate twofold increase in mitochondrial width in control cells under staurosporine treatment (similar to our immunofluorescence results), while mitochondrial widths in LACTB KD cells do not change significantly (Fig. 4H). In addition, there is a significant reduction in the number of cristae in control cell mitochondria upon apoptotic induction, a feature absent in LACTB KD cells (Fig. 4I). We also used a recently developed fluorescent dye that stains cristae, PKmito Orange FX (*38*). Consistent with our EM and immunofluorescence data, control cell cristae exhibit extensive remodeling following apoptotic induction with staurosporine or Q-ABT-S. In contrast, cristae in LACTB KD cells retain their structure, resembling those in untreated samples (fig. S7, C to G).

**Fig. 5.**
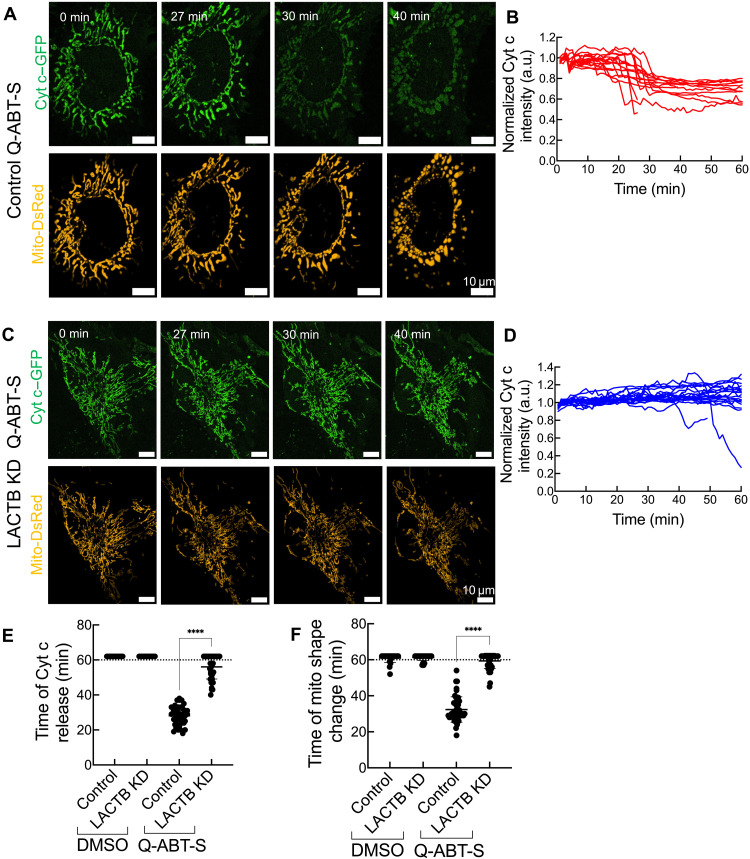
LACTB depletion slows cytochrome c release and mitochondrial remodeling kinetics upon apoptotic stimulation. (**A**) Live-cell imaging of cytochrome c release (Cyt c–GFP) and mitochondrial shape changes (Mito-DsRed) in HeLa cells treated with ABT-S in the presence of the pan-caspase inhibitor Q-VD-OPh (20 μM, 1-hour preincubation). (**B**) Quantification of cytochrome c release kinetics in control siRNA-treated cells treated with Q-ABT-S. *N* = 3 independent experiments; *N*_cells_ = 16. (**C**) Live-cell imaging of cytochrome c release and mitochondrial shape changes in LACTB KD cells treated with Q-ABT-S and Q-VD-OPh [as in (A)]. (**D**) Quantification of cytochrome c release kinetics in LACTB KD cells treated with Q-ABT-S. *N* = 3 independent experiments; *N*_cells_ = 18. (**E**) Quantification showing the onset of cytochrome c release in control and LACTB KD cells under DMSO and Q-ABT-S treatment. *N* = 3 independent experiments; *N*_cells_ = 39. (**F**) Quantification showing the onset of mitochondrial shape changes in control and LACTB KD cells under DMSO and ABT-S treatment. *N* = 3 independent experiments; *N*_cells_ = 39. For [(E) and (F)], all quantifications were performed in a blinded manner. No cytochrome c release or mitochondrial shape changes were observed for data points above the dotted line. Error bars represent means ± SD. *N* indicates the number of independent experiments. One-way ANOVA, ****P < 0.0001.

To investigate the kinetics of mitochondrial shape change in live cells during apoptosis, we transfected a matrix marker (Mito-DsRed) into control and LACTB KD cells. We also monitored cytochrome c release kinetics using a previously described cytochrome c–GFP construct (*23*, *39*). Prior to imaging, cells were treated with a pan-caspase inhibitor (Q-VD-OPh) for 1 hour and then mounted on the microscope and stimulated with ABT-S. In control cells, cytochrome c release occurs at 28 ± 5.4 min poststimulation (Fig. 5, A, B, and E, and movie S1), followed by mitochondrial shape changes at 32 ± 7.1 min (Fig. 5, A and F, and movie S2). In contrast, ~70% of LACTB KD cells display no cytochrome c release or shape change over the 60-min viewing period, and the ~30% of cells that do show an effect exhibit a significant delay in both cytochrome c release and mitochondrial remodeling (Fig. 5, C to F, and movies S3 and S4).

We also used live-cell analysis to test whether LACTB influences mitochondrial depolarization upon apoptosis induction. In control cells, Q-ABT-S induces abrupt mitochondrial depolarization, as assessed by tetramethylrhodamine ethyl ester perchlorate (TMRE) staining (fig. S8, A, C, E, and G, and movie S5). The onset of depolarization is variable, occurring as early as 12 min poststimulus and as late as 34 min (fig. S8E). In all cases, however, depolarization is abrupt after onset, with complete depolarization within ~4 to 5 min. In contrast, 12% of LACTB KD cells undergo any depolarization over the 60-min imaging period, with the onset of the few that do being >45 min poststimulus (fig. S8, B, D, F, and H, and movie S6). These results support the possibility that LACTB contributes to mitochondrial remodeling during apoptosis.

Mitochondrial membrane remodeling can also be induced by carbonyl cyanide m-chlorophenyl hydrazone (CCCP), a potent mitochondrial depolarizer (*40*, *41*). We tested whether LACTB KD alters CCCP-induced mitochondrial remodeling. There is no difference in mitochondrial remodeling between control and LACTB KD cells upon CCCP treatment (fig. S9, A and B).

Together, these results demonstrate that LACTB plays a critical role in regulating mitochondrial morphology and cristae remodeling during apoptosis. The fact that LACTB KD does not alter remodeling caused by CCCP suggests that LACTB’s role is function-specific, rather than a general effect.

### LACTB’s protease activity and membrane binding are required for mitochondrial remodeling

LACTB is a serine protease and is also capable of binding membrane (*14*, *42*). To test this, we used the PiggyBac system to generate HeLa cell lines stably overexpressing wild-type (WT) LACTB, a protease-deficient mutant (SISK→AISK) (*42*), or a membrane-binding mutant (ΔAH) (*42*). Expression levels and localization to mitochondria were confirmed by immunoblotting (fig. S10, G and H). Under basal conditions (no apoptotic stimulus), OE of WT LACTB or its mutants do not alter mitochondrial morphology, as measured by immunofluorescence staining for the OMM protein Tom20 and the IMM protein ATP synthase β (fig. S10, A to F).

**Fig. 6.**
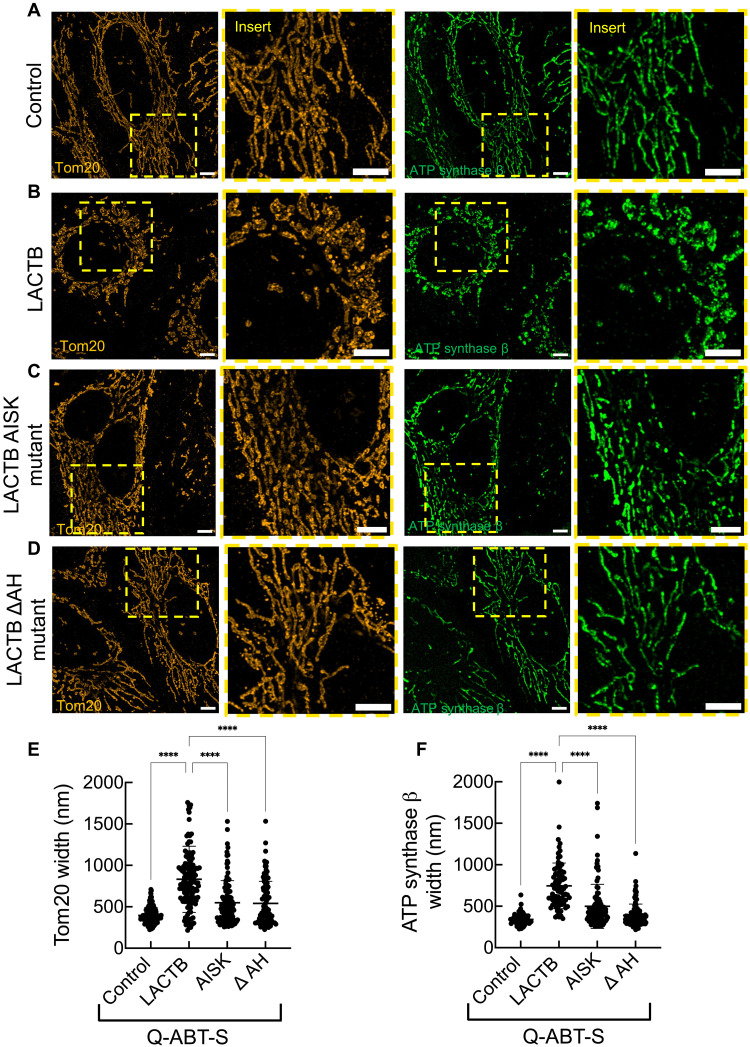
LACTB modulates mitochondrial morphology under apoptosis-inducing conditions. (**A** to **D**) Immunofluorescence staining of Tom20 (orange) and ATP synthase β (green) in control (A), LACTB OE (B), LACTB active-site mutant (AISK) (C), and LACTB amphipathic helix mutant (ΔAH; putative membrane-binding mutant) (D) cells treated with ABT-S (10 μM ABT-737 and 2 μM S63845) for 20 min in the presence of the pan-caspase inhibitor Q-VD-OPh (20 μM, 1-hour preincubation) Scale bars, 5 μm. (**E** and **F**) Quantification of mitochondrial width based on Tom20 and ATP synthase β fluorescence under ABT-S treatment. Data represent *N* = 2 independent experiments; *N*_mito_ ≥ 104. Error bars indicate means ± SD. One-way ANOVA, ****P < 0.0001.

To assess LACTB’s role in apoptosis-induced mitochondrial remodeling, we treated cells with Q-ABT-S for 20 min. At this early time point, prior to overt morphological changes in most control cells, WT LACTB OE significantly increases mitochondrial width compared to vector control cells (Fig. 6, A, B, E, and F). In contrast, cells expressing either the protease-dead (AISK) or membrane binding–deficient (ΔAH) mutant fail to show such remodeling (Fig. 6, C to F). These results suggest that both the protease activity and membrane-binding ability of LACTB are required for its function in promoting mitochondrial shape changes during apoptosis.

### LACTB-mediated mitochondrial remodeling is independent of Opa1 processing

The proteolytic processing of OPA1 has previously been implicated in IMM remodeling during apoptosis (*11*). To investigate whether the resistance to mitochondrial shape changes observed in LACTB KD cells was due to changes in OPA1 levels or processing, we examined OPA1 by Western blotting. A slight increase in OPA1 levels occurs in LACTB KD cells compared to controls (fig. S11A). However, OPA1 processing to the short form occurs normally in LACTB KD cells following treatment with Q-ABT-S (fig. S11A). We also tested OPA1 processing during staurosporine-induced apoptosis. No clear changes in OPA1 processing occur in either control or LACTB KD cells following 1 hour of staurosporine treatment (fig. S11A), a time period sufficient to induce cytochrome c release and mitochondrial shape changes. We also tested the effect of Opa1 KD on cytochrome c–GFP release upon Q-ABT-S treatment. Consistent with previous report (*39*), Opa1 KD does not significantly alter the kinetics of cytochrome c release (fig. S11, B to D). These results suggest that LACTB mediates mitochondrial remodeling in a manner distinct from Opa1.

### LACTB does not alter Drp1 recruitment to mitochondria during apoptosis

Since dynamin-related protein 1 (Drp1) is known to interact with BAX and facilitate its recruitment to mitochondria during apoptosis (*43*), we next investigated whether LACTB influences Drp1 recruitment under apoptotic conditions. Immunofluorescence staining for Drp1 in control HeLa cells reveals increased Drp1 recruitment to mitochondria following 30 min of Q-ABT-S treatment (fig. S12, A to C). LACTB KD causes no significant difference in Drp1 mitochondrial recruitment (fig. S12, A to C), suggesting that LACTB does not regulate Drp1 localization during apoptosis.

We also compared the effects of LACTB KD versus Drp1 KD on mitochondrial membrane potential and mitochondrial area. To assess mitochondrial membrane potential, we used TMRE. LACTB KD causes a 22% reduction in mitochondrial membrane potential, although the decrease is less pronounced than that caused by Drp1 KD (35%; fig. S13, A to E). In terms of mitochondrial area, LACTB KD causes a 1.6-fold increase while Drp1 KD causes a 3.4-fold increase relative to control cells (fig. S13, F and G). In combination with the lack of effect of LACTB KD on the widths of mitochondria in untreated cells (discussed earlier), these results suggest that LACTB KD has minor effects on mitochondria in the absence of apoptotic stimuli.

### LACTB binds and remodels cardiolipin-containing curved membranes

We have shown that LACTB (i) acts in apoptosis by facilitating release of cytochrome c and other factors from mitochondria, (ii) colocalizes with ATP synthase, and (iii) plays a role in mitochondrial morphology changes during apoptosis. Given that LACTB has previously been reported to bind cardiolipin-containing membranes (*42*), we investigated whether LACTB actively participates in membrane remodeling. We tested the binding of purified LACTB-GFP (fig. S14, A and B) to supported lipid membranes, using a system in which membrane nanotubes are pulled from vesicles, with a supported lipid bilayer (SLB) at the source where lipids are spotted (*44*, *45*). These nanotubes mimic cristae dimensions (20 to 60 nm in diameter) (fig. S14C). This assay offers the ability to monitor a range of membrane curvatures simultaneously, from the planar SLB to nanotubes of varying diameter.

**Fig. 7.**
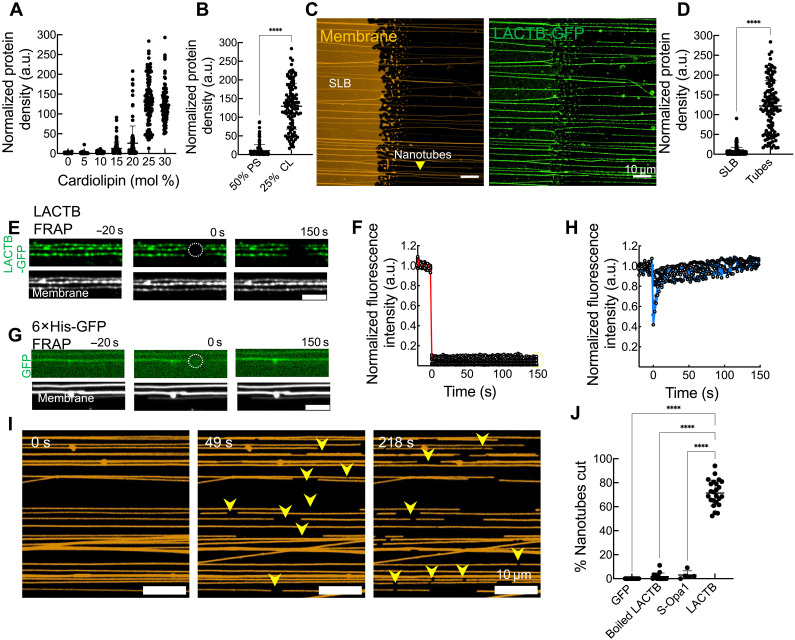
LACTB preferentially binds and remodels curved membranes containing cardiolipin. (**A**) Binding of 1 μM LACTB-GFP to membrane nanotubes in response to increasing cardiolipin concentration. Lipid mixtures contained DOPC and varying levels of cardiolipin, with 0.5 mol % rhodamine-labeled phosphatidylethanolamine (RhPE) for visualization. *N*_tubes_ ≥ 80. (**B**) Quantification of 1 μM LACTB-GFP binding to nanotubes containing either 25 mol % cardiolipin or 50 mol % phosphatidylserine. *N*_tubes_ ≥ 103. (**C** and **D**) Representative micrographs and quantification of 1 μM LACTB-GFP (green) binding on nanotubes compared to SLBs. Lipid composition: DOPC:CL:RhPE (74.5:25:0.5 mol %). *N* = 3; *N*_SLB_ = 155; *N*_tubes_ = 119. (**E** and **F**) Representative images (E) and fluorescence recovery curves (F) showing FRAP kinetics of LACTB-GFP on nanotubes containing 25 mol % cardiolipin. *N* = 2; *N*_tubes_ = 15. Scale bar, 5 μm. (**G** and **H**) Representative images (G) and fluorescence recovery curves (H) showing the FRAP kinetics of 6×His-GFP alone on membrane nanotubes containing 5 mol % Ni-NTA. *N* = 1; *N*_tubes_ = 6. Scale bar, 5 μm. (**I**) Stills from a time-lapse movie showing the membrane marker RhPE upon LACTB (1 μM) addition to nanotubes containing 25 mol % cardiolipin for the indicated times. Yellow arrowheads indicate sites of fission on the nanotubes. (**J**) Quantification of nanotube fission after incubation with 1 μM LACTB, S-Opa1, boiled LACTB, or GFP for 10 min. Each data point represents an entire field containing 10 to 15 nanotubes. *N* = 3 independent experiments; *N*_tubes_ ≥ 104. Error bars represent means ± SD. Mann-Whitney test, ****P < 0.0001.

Efficient membrane binding by LACTB-GFP requires ~25 mol % cardiolipin (Fig. 7A and fig. S14D). This requirement is not solely due to cardiolipin’s negative charge, as equivalent charge density of phosphatidylserine is insufficient for binding (Fig. 7B and fig. S14D). LACTB-GFP binding occurs mainly on nanotubes, suggesting that LACTB exhibits a strong preference for curved membranes (Fig. 7, C and D).

Dynamic monitoring of LACTB-GFP binding to nanotubes reveals punctate assembly, with punctae nucleating at specific sites on the tubes (fig. S14E). The assembly process follows a half-time of 52.9 ± 3 s (fig. S14F). Once formed, these “scaffolds” do not exchange with free LACTB-GFP in solution, as demonstrated by fluorescence recovery after photobleaching (FRAP) analysis (Fig. 7, E and F). This behavior contrasts with 6× histidine (His)–tagged GFP binding to nanotubes containing Ni–nitrilotriacetic acid (NTA) lipid, which displays uniform binding and rapid recovery after FRAP (fig. S14E and Fig. 7, G and H).

LACTB binding also induces nanotube fission, observed in real time (Fig. 7I, movie S7, and fig. S15A). These fission events require properly folded LACTB, as neither boiled LACTB nor soluble GFP cause nanotube fission (Fig. 7J and fig. S15, B and C). As an independent control, we tested whether the short form of Opa1 (S-Opa1) could remodel cardiolipin-containing membrane nanotubes in the presence of guanosine 5′-triphosphate and MgCl_2_. Unlike LACTB, S-Opa1 does not sever nanotubes under these conditions, despite interacting with the membrane (Fig. 7J and fig. S15, D to F). Montages of nanotubes undergoing fission in the presence of LACTB reveal that a distinct drop in fluorescence intensity occurs at the fission site prior to fission, suggesting that LACTB induces membrane constriction en route to fission (fig. S15, G and H). Using kymograph analysis, we captured membrane intermediates preceding fission in some instances (fig. S15, I and J) but not in others (fig. S15K). Together, these findings demonstrate that LACTB preferentially binds curved membrane surfaces in a punctate manner and is capable of inducing membrane constriction as well as fission.

## DISCUSSION

The mechanisms underlying IMM remodeling during apoptosis as well as their role in cell death remain controversial (*1*, *8*, *46*, *47*). Previous work has shown that the IMM dynamin Opa1 might play an inhibitory role in this process by maintaining cristae architecture, thereby delaying cytochrome c release (*11*). Several mechanisms might relieve this Opa1-mediated inhibition, including BH3-only proteins (*9*–*12*), the mitochondrial fission machinery (*48*, *49*), and proteolytic cleavage by the IMM protease metalloendopeptidase (OMA1) (*50*, *51*). Conversely, the IMM protease PARL (presenilin-associated rhomboid-like protein) has been reported to cleave Opa1 in a manner that enhances its antiapoptotic activity (*9*, *11*), although another study implicates PARL as proapoptotic via a different mechanism (*52*). Moreover, it remains unclear whether IMM remodeling is essential for apoptosis, as one study found that such remodeling occurs after cytochrome c release during etoposide-induced apoptosis (*37*).

Our work reveals three fundamental findings: (i) The IMS protein LACTB enhances apoptosis through promoting mitochondrial protein release, (ii) LACTB is also required for mitochondrial remodeling during apoptosis, and (iii) purified LACTB can directly remodel phospholipid membrane nanotubes. LACTB function does not appear to be directly related to mitochondrial BAX or Drp1 recruitment but to mitochondrial remodeling. One possible explanation for our results is that altered BAX/BAK oligomerization after mitochondrial recruitment could result in the formation of smaller pores in the outer mitochondrial membrane. While we have not directly tested this possibility here, the predominant effect of LACTB KD is the suppression of apoptosis-induced mitochondrial reorganization, suggesting that any impact on pore formation may occur downstream of these structural changes.

The fact that LACTB KD inhibits apoptosis-induced mitochondrial remodeling, but not remodeling caused by CCCP, suggests that LACTB’s function is purpose-specific rather than as a general remodeling factor. In addition, altering LACTB levels by KD do not change Opa1 processing, suggesting that LACTB’s effects are independent of Opa1-induced mitochondrial remodeling. We find that Opa1 KD accelerates cell death, similar to previous reports (*11*, *39*). This observation highlights that OPA1 may exert a protective effect under apoptotic stress, in contrast to LACTB, which promotes mitochondrial remodeling and facilitates apoptosis progression.

One possibility is that LACTB influences apoptosis indirectly by affecting mitochondrial fitness, thereby altering the cell’s susceptibility to apoptotic stimuli. LACTB KD leads to a decrease in mitochondrial membrane potential and an increase in mitochondrial length. However, these effects are less pronounced than those observed with Drp1 KD. Moreover, in the absence of apoptotic stimulation, LACTB KD has minimal impact on cristae morphology or mitochondrial width. Together with published results showing a minor effect of LACTB knockout on basal oxygen consumption (*30*), these findings support a model in which LACTB plays a more direct role in apoptosis, rather than exerting its effects solely through general perturbations in mitochondrial function.

Our biophysical studies reveal that LACTB preferentially binds and remodels curved membranes enriched in cardiolipin, raising the possibility of a direct role in mitochondrial membrane remodeling during apoptosis. These findings establish LACTB as a unique membrane remodeling factor in mitochondrial dynamics during apoptosis, distinct from mitochondrial dynamin proteins like Opa1, Drp1, and mitofusins (*45*).

Our live-cell assays indicate that apoptotic cytochrome c release precedes overt changes in mitochondrial morphology, consistent with previous findings (*37*). This raises the question of whether these two processes are functionally connected. LACTB KD strongly suppresses both cytochrome c release and mitochondrial morphological changes, suggesting a potential link. One possibility is that subtle morphological alterations required for cytochrome c release from cristae fall below the resolution of our imaging system, with the larger-scale changes we detect occurring subsequently. Alternatively, these events may both depend on LACTB but proceed through distinct, independent mechanisms.

Another question is why would LACTB-mediated IMM remodeling affect release of proteins that are freely soluble in the IMS. While cytochrome c is known to be cristae-bound, and AIF is thought to be an integral membrane protein in the IMM, proteins such as SMAC/DIABLO and HTRA2 are commonly described as freely soluble within the IMS (*53*). However, several findings challenge this assumption, reporting that a significant fraction of these proteins remain mitochondria-associated under basal or apoptotic conditions, requiring alkaline pH or stronger stimuli for release (*52*, *54*, *55*). Localization studies have also implicated HTRA2’s proximity to IMM subdomains and components such as mitochondrial contact site and cristae organizing system (MICOS complex), supporting a model in which IMM remodeling may be necessary for its release (*56*, *57*). Thus, our findings that LACTB modulates IMM architecture may provide mechanistic insight into how the release of such IMS proteins is coordinated during apoptosis.

We also found that OE of LACTB accelerates mitochondrial morphology changes during apoptosis. Notably, both the protease active-site mutant and the membrane-binding mutant exhibit a significant reduction in mitochondrial width changes compared to WT LACTB at an early time point after apoptotic induction. These findings suggest that both LACTB’s enzymatic activity and membrane association are essential for its role in mitochondrial membrane remodeling during apoptosis. At present, we do not know how these mutants behave at later apoptotic stages. Addressing this question, as well as examining mutant function in the LACTB KD background, will be important for clarifying the precise mechanisms underlying LACTB’s role.

While one potential mechanism for LACTB-mediated IMM remodeling is its direct function as a membrane remodeling protein, another possibility is that LACTB stimulates membrane remodeling through other proteins. A previously proposed mechanism for LACTB function is through proteolysis of the PISD protein, an enzyme that converts phosphatidylserine to phosphatidylethanolamine (*14*). While we show that PISD processing is not altered by LACTB KD or OE, other proteins might be LACTB substrates during apoptosis, with one candidate being phospholipase A2 group VI (*30*). As another possibility, LACTB could mediate contact between inner and outer mitochondrial membranes, facilitating cardiolipin transfer to the outer membrane, a process critical for BAX activation (*58*–*60*). Future studies will investigate the molecular mechanism of LACTB’s apoptotic role in more detail. It will also be interesting to determine whether LACTB plays a role in mitochondrially derived vesicle/mitochondrially derived compartment assembly (*61*, *62*) or in mitochondrial nucleic acid release upstream of inflammatory activation through cGAS-STING (cyclic GMP-AMP synthase-Stimulator of Interferon Genes) (*63*, *64*) and other pathways.

Our results suggest that LACTB functions as a proapoptotic factor but is not strictly required for apoptosis. LACTB KD delays key apoptotic events, including caspase-3 activation, PARP cleavage, and cell death, yet these processes are ultimately still triggered in response to apoptotic stimuli. Conversely, LACTB OE accelerates cell death. These findings suggest that while LACTB facilitates the efficiency and kinetics of apoptosis, alternative mitochondrial remodeling mechanisms may compensate in its absence.

Our identification of a role for LACTB in mitochondrial protein release does not rule out other tumor-suppressing functions. It is possible that LACTB is itself released from mitochondria and plays additional roles through its previously reported interactions with p53 (*15*), PP1A (*16*), and components of the PI3K/AKT signaling pathway (*20*).

Our work establishes LACTB as an important regulator of mitochondrial morphology during apoptosis. By identifying its curvature-specific membrane remodeling activity and its distinct role in apoptotic signaling, we provide a foundation for exploring LACTB’s therapeutic potential, particularly in diseases where mitochondrial dynamics and apoptosis are dysregulated.

## MATERIALS AND METHODS

### Cell culture

Human cervical cancer (HeLa cells), osteosarcoma (U2-OS) cells, and murine melanoma (B16-F10) cells were obtained from the American Type Culture Collection. Cells were cultured in Dulbecco’s modified Eagle’s medium (DMEM; Corning, 10-013-CV) supplemented with 10% fetal bovine serum (FBS; Sigma-Aldrich, F4135) at 37°C in a humidified atmosphere containing 5% CO_2_. Mycoplasma contamination was monitored every 3 months using the MycoStrip Mycoplasma Detection Kit (InvivoGen, rep-mys-50). All lines were maintained for no more than 30 passages.

### Small interfering RNA knockdown

Oligonucleotides for small interfering RNA (siRNA)–mediated silencing were synthesized by Integrated DNA Technologies (IDT). For human LACTB, sequence targeting exon 5 (3′ untranslated region) was 5′-ACUUGGAUAUGCUGACGACUGUGCA-3′. For mouse LACTB, sequences targeting coding DNA sequence (CDS) exon 3 was 5′-AAAAGGUUUCUGUCACAACAAGATT-3′. Human OPA1 was targeted using sequence to exons 19 to 22: 5′-CCACAGUGGAUAUCAAGCUUAAACA-3′. Human Drp1 targeting CDS exon 8 was 5′-GCCAGCUAGAUAUUAACAACAAGAA-3′. Negative control siRNA sequence was 5′-CGUUAAUCGCGUAUAAUACGCGUAU-3′. Cells were seeded at 7.5 × 10^4^ per well in six-well dishes and cultured overnight in 2 ml of medium. The following day, transfection mixes were prepared by incubating 63 pmol of each siRNA with 50 μl of Opti-MEM (Gibco, 31985-070) and 2 μl of RNAiMAX (Thermo Fisher Scientific, 13778) with 100 μl of Opti-MEM separately for 10 min. The two solutions were then combined and incubated for an additional 20 min. Medium was replaced with 1 ml of fresh medium, and 150 μl of the transfection mix was added dropwise. After 10 hours, medium was replaced with 2 ml of medium. Transfections were repeated 48 hours later. At 72 hours after the initial transfection, cells were replated at seeding density of 2 × 10^5^ cells onto fibronectin-coated (Sigma-Aldrich, F1141-1MG) MatTek dishes (MatTek, P35G-1.5-14-C) and processed for fixation or live cells microscopy.

### Plasmids and transfections

Mito-GFP was purchased from Clontech (pAcGFP1-Mito, 632432) and contains the mitochondrial targeting sequence derived from the precursor of subunit VIII of human cytochrome c oxidase. Mito-DsRed was previously described (*65*). Tom20-GFP was previously described (*40*). Cytochrome C–GFP was obtained from D. Green (Addgene plasmid #41182). The plasmid pMSCV-OPA1 was a gift from D. Chan (Addgene plasmid #26047). A truncated form of OPA1 encoding amino acids 195 to 960 (S-Opa1) (*66*) was amplified by polymerase chain reaction (PCR) from this construct, and a C-terminal Strep II tag was added during cloning to facilitate purification. The PCR product was cloned into the bacterial expression vector pET-16b using Xho I and Bam HI restriction sites. The Super PiggyBac Transposase was purchased from System Biosciences LLC (PB210PA-1), and the PiggyBac Dual Promoter (PB513B-1) was purchased from Life Science Market (empty plasmid used as control for OE). The human LACTB coding sequence was PCR-amplified from a sequence-verified cDNA clone (accession: BC067288; clone ID: 30366453; Dharmacon, MHS6278-202804693) and inserted into the PiggyBac Dual Promoter vector using Xba I and Not I restriction sites and into the enhanced GFP–N1 mammalian expression vector (Clontech) using Xho I and Kpn I restriction sites with 2× Strep II tag at C terminus using PCR. Two LACTB mutants were generated: (i) a protease-inactive mutant by substituting residues 164 to 167 (SISK) with AISK and (ii) a membrane-binding mutant by deleting the lysine-rich flexible loop region (amino acids 224 to 291) from the WT LACTB sequence (*42*). Both mutant sequences were cloned into the PiggyBac Dual Promoter expression vector using Xba I and Not I restriction sites. All constructs were verified by Sanger sequencing prior to use. Plasmid transfections were initiated by seeding cells at 4 × 10^5^ cells per well in 35-mm dishes ~16 hours prior to transfection. Transfections were carried out in Opti-MEM medium (Gibco, 31985062) using Lipofectamine 2000 (Invitrogen, 11668) according to the manufacturer’s protocol. Posttransfection, cells were trypsinized and replated onto fibronectin-coated glass-bottom dishes (MatTek Corporation, P35G-1.5-14-C) at a density of ~1 × 10^5^ cells per well. Imaging was performed ~16 to 24 hours after transfection. For preparation of stable cell lines by the PiggyBac system, transfections were performed following the manufacturer’s protocol (System Biosciences LLC), and puromycin selection (2 μg/ml) was applied at 24 hours posttransfection for 3 to 5 days to establish stably transfected cell lines.

### Antibodies

Details of all antibodies used in this study, including their sources, catalog numbers, and dilutions, are provided in table S1.

### Drug treatments

Staurosporine (Research Product International, S63500-0.0001; referred to as STS in figures) was used at 1 mM. The combination of ABT-737 (10 μM; ApexBio, A8193) and S63845 (2 μM; ApexBio, A8737) was collectively referred to as ABT-S in figures. CCCP (20 μM for 20 min; Sigma-Aldrich, C2759). For experiments involving the pan-caspase inhibitor Q-VD-OPh (20 μM; ApexBio, A1901), cells were preincubated for 1 hour prior to ABT-S treatment (referred to as Q-ABT-S in figures). All treatments were performed in prewarmed medium and incubated at 37°C with 5% CO_2_ for the indicated durations.

### SRB assay

Assay was performed as per reported protocol (*24*). Briefly, 1 × 10^4^ cells were plated per well (100 ml) in a 96-well plate and incubated overnight. The next day, 100 μl of medium containing either the drug of interest or DMSO was added to each well. At the end of the treatment, medium was aspirated; cells were fixed by adding 100 μl of 10% trichloroacetic acid (TCA; Sigma-Aldrich, T6399) per well and incubating for 30 min at 4°C. The TCA was then removed, and the wells were washed five times with Milli-Q water using a multichannel pipette. Plates were air-dried completely before staining each well with 100 μl of 0.4% SRB (Sigma-Aldrich, 230162) in 1% acetic acid for 10 min at room temperature. Excess dye was removed by washing the wells five times with 1% acetic acid, followed by air drying. The bound dye was solubilized by adding 100 μl of 10 mM tris-HCl (pH 8.0) to each well and gently shaking for 5 min. Absorbance was measured at 520 nm using an M-1000 plate reader (Tecan Inc.) through i-control software (version1.11). Each data point in the SRB assay graph represents an individual well (i.e., technical replicates), and it measures relative protein content*.*

### Annexin V staining

Cells were harvested using 0.05% trypsin, washed with 1× phosphate-buffered saline (PBS; Corning, 21-040-CM), counted, and normalized to a concentration of 1 × 10^6^ cells/ml. Apoptosis and cell death markers were stained using PE Annexin V Apoptosis Detection Kit with 7-AAD (BioLegend, 640934) following the manufacturer’s protocol. Cells were washed twice with staining buffer [PBS with 2% newborn calf serum (Gibco, 26010074)] and resuspended in 50 μl of Annexin V Binding Buffer per sample. Two microliters of PE Annexin V and 2 μl of 7-AAD Viability Staining Solution were added. Cells were gently mixed and incubated for 15 min at room temperature in the dark. Following incubation, 150 μl of Annexin V Binding Buffer was added to achieve a final volume of 200 μl. Flow cytometry was performed immediately using a CytoFLEX S flow cytometer (Beckman Coulter), and data were analyzed with FlowJo v10 software (BD Biosciences).

### Cytosol isolation

Protocol adapted from Bossy-Wetzel *et al.* (*31*). Cells were trypsinized and resuspended in DMEM supplemented with 10% FBS to neutralize the trypsin. The cells were then washed with 1× PBS to remove excess medium and pelleted by centrifugation at 200*g* for 10 min at 4°C. The resulting pellet was resuspended in 500 μl of extraction buffer [220 mM mannitol, 68 mM sucrose, 20 mM Hepes (pH 7.4), 50 mM KCl, 2 mM MgCl_2_, 1 mM dithiothreitol (DTT), and protease inhibitors] and incubated on ice for 30 min. Subsequently, the cells were homogenized for 20 strokes using a prechilled Dounce homogenizer (Wheaton Dura-Grind, 357574) and centrifuged at 14,000*g* for 15 min at 4°C. The pellet (nuclear, endoplasmic reticulum, and mitochondrial fraction) and the supernatant (cytosolic fraction) were separated. Protein concentration in the cytosolic fraction was measured using the bicinchoninic acid assay (Thermo Fisher Scientific, 23225), and samples were prepared with 4× SDS–polyacrylamide gel electrophoresis (SDS-PAGE) sample buffer diluted to 1×.

### Western blot

For cell extracts, lysate preparation involved washing confluent cells grown in 35-mm dishes three times with 1×PBS and lysing them in ∼400 μl of 1× sample buffer, consisting of 50 mM tris-HCl (pH 6.8), 2 mM EDTA, 20% glycerol, 0.8% SDS, 0.02% bromophenol blue, 1000 mM NaCl, and 4 M urea. Lysates were heated at 95°C for 10 min, and genomic DNA was sheared by sonicating for 10-min in bath sonicator. For cytosolic and mitochondrial fractions, samples were prepared as described above. Proteins were resolved using SDS-PAGE and transferred onto polyvinylidene difluoride membranes (Millipore). Blocking was performed in tris-buffered saline with Tween 20 (TBS-T) buffer [20 mM tris-HCl (pH 7.6), 136 mM NaCl, and 0.1% Tween 20] containing 3% bovine serum albumin for 1 hour at room temperature, followed by overnight incubation with primary antibodies at 4°C. After three washes with TBS-T, membranes were incubated for 1 hour at room temperature with horseradish peroxidase–conjugated secondary antibodies. Excess secondary antibodies were removed by three additional TBS-T washes. Chemiluminescence signals were detected using ECL Prime Western Blotting Detection Reagent (Cytiva Amersham, 45-002-40) and recorded with an ECL chemocam imager (SYNGENE G:BOX Chemi XRQ). For fluorescence-based detection, membranes were incubated with IRDye 680 goat anti-mouse or IRDye 800CW goat anti-rabbit secondary antibodies for 1 hour at room temperature, and signals were visualized using the LI-COR Odyssey CLx imaging system. Information regarding primary and secondary antibodies is provided in table S1.

### Immunofluorescence staining

Cells were plated onto fibronectin-coated MatTek dishes 16 hours prior to fixation and staining. Treatments were performed at 37°C and 5% CO_2_, after which cells were washed twice with PBS and fixed in either 1% glutaraldehyde (EMS, 16020) for 10 min or 4% prewarmed paraformaldehyde (EMS, 15170) prepared in PBS for 20 min. Fixed cells were permeabilized using 0.1% Triton X-100 in PBS for 10 min and blocked in PBS containing 10% calf serum for 1 hour. Cells were stained with the appropriate primary antibody (table S1) diluted in PBS containing 1% calf serum for 1.5 hours, followed by three PBS washes and incubation with the corresponding secondary antibodies (table S1) and 4′,6-diamidino-2-phenylindole (DAPI; Calbiochem, 268298) for nuclear staining in PBS containing 1% calf serum for 1 hour. After washing in PBS, imaging was conducted in PBS using either Dragonfly spinning disk or Airyscan.

### Airyscan microscopy

Airyscan imaging was conducted using an LSM 880 confocal microscope equipped with a 100×/1.4 numerical aperture (NA) Apochromat oil objective and Airyscan detectors (Carl Zeiss Microscopy). Imaging was performed with a 488-nm laser and a band pass (BP) 420 to 480/BP 495 to 620 filter for fluorescein-labeled secondary antibodies, and a 561-nm laser with a BP 495 to 550/long pass 570 filter for Texas Red–labeled secondary antibodies. *Z*-stack images were acquired from the basal to apical regions with a step size of 0.2 μm. Raw image data were processed using the Airyscan processing feature in Zen Black software (Carl Zeiss, version 2.3). Maximum intensity projections were generated from *z*-stacks, and background subtraction was performed in ImageJ Fiji software using the rolling ball algorithm (radius: 20).

### Live cell microscopy using spinning disk confocal microscope

Live-cell imaging was performed in DMEM (Corning, 10-013-CV) supplemented with 10% FBS (Sigma-Aldrich, F4135). Approximately 2 × 10^5^ cells were plated onto fibronectin-coated MatTek dishes 16 hours before imaging. The medium was pre-equilibrated at 37°C and 5% CO_2_ prior to use. Cells were preincubated for an hour prior to imaging with the pan-caspase inhibitor Q-VD-OPh (20 μM; ApexBio, A1901) in DMEM supplemented with 10% FBS.

Imaging was conducted using a Dragonfly 302 spinning disk confocal system (Andor Technology) on a Nikon Ti-E microscope base. The system was equipped with an iXon Ultra 888 EMCCD camera, a Zyla 4.2 Mpixel sCMOS camera, and a Tokai Hit stage-top incubator maintained at 37°C. Illumination was provided by a solid-state 405 smart diode laser (100 mW), a solid-state 560 OPSL smart laser (50 mW), and a solid-state 637 OPSL smart laser (140 mW). For drug treatment experiments, the compound was added at the start of the fourth image frame (~4 min after imaging began; time interval set at 1 min), and imaging continued for a total duration of 1 hour. The CFI Plan Apochromat Lambda 100×/1.45 NA oil objective (Nikon, MRD01905) was used for live-cell drug treatment assays as well as fixed cell imaging. The CFI Plan Apochromat 60×/1.4 NA oil immersion objective was used for imaging during BAX localization experiments. Image acquisition was carried out using Fusion software (Andor Technology, version 2.0.0.15).

### PKmito Orange FX staining

PKmito Orange FX (Cytoskeleton, Inc., CY-SC054) staining was conducted following the manufacturer’s protocol. Briefly, control and LACTB KD cells were seeded at an equal density (2 × 10^5^ cells) onto fibronectin-coated MatTek dishes 16 hours prior to imaging. On the day of imaging, cells were preincubated with medium containing 500 nM PKmito Orange FX at 37°C in a humidified atmosphere with 5% CO_2_ for 1 hour. Drug treatments were carried out in the presence of 500 nM PKmito Orange FX for the indicated durations.

Following treatment, cells were washed three times with PBS and fixed with prewarmed 2.5% glutaraldehyde for a minimum of 30 min. The cells were then washed three more times with PBS, and DNA staining was performed by adding 0.1 mg/liter (w/v) DAPI to the first wash to label the nucleus. Imaging was performed using Airyscan microscope.

### Mitochondrial membrane potential staining using TMRE

For mitochondrial membrane potential detection using TMRE (Sigma-Aldrich, 87917), cells were incubated with 30 nM TMRE (from a 30 μM stock in DMSO) for 10 min at 37°C in DMEM supplemented with 10% FBS, prior to apoptosis induction.

### Liposome sedimentation assay

All lipids were obtained from Avanti Polar Lipids. Liposomes were prepared at a final composition of 25 mol % cardiolipin and 75 mol % 1,2-dioleoyl-sn-glycero-3-phosphocholine (DOPC), with a total lipid concentration of 1 mM. Lipids were mixed in the specified molar ratios in a clean glass tube, dried under high vacuum, and rehydrated in 1× PBS at 50°C for 1 hour. To generate small unilamellar vesicles, the lipid suspension was sonicated at 20% amplitude, using a 1-s on/1-s off cycle for 5 min.

For the liposome sedimentation assay, S-Opa1 (1.5 μM) was incubated with or without liposomes (100 μM) in 1× PBS for 30 min at room temperature. Samples were centrifuged at 100,000*g*, and the resulting pellet (liposome-bound fraction) and supernatant (unbound fraction) were analyzed by 10% SDS-PAGE, followed by staining with Coomassie Brilliant Blue.

### Thin-section EM

Control and LACTB KD cells were seeded at a density of 3 × 10^5^ cells/ml onto fibronectin-coated MatTek dishes 16 hours prior to fixation. On the day of fixation, cells were treated with staurosporine or DMSO (control) and incubated for 1 hour. Fresh fixative containing 0.1 M sodium cacodylate buffer, 2% glutaraldehyde, and 3.2% paraformaldehyde in Milli-Q water was prepared within an hour before use and warmed to 37°C in a tissue culture bath. Medium was aspirated from the dish, and 2 ml of warm fixative was added immediately without prior washing. After 15 min, the fixative was replaced with 1.5 ml of fresh fixative from the same batch, and cells were incubated at room temperature for 1 hour. The dishes were sealed with parafilm and stored in an airtight container for subsequent processing. Postfixation was carried out with 1% osmium tetroxide in 0.1 M sodium cacodylate buffer (pH 7.2) for 1 hour on ice in the dark, followed by rinsing twice with 0.1 M sodium cacodylate buffer for 10 min each at room temperature. Samples were incubated overnight in 2% aqueous uranyl acetate at room temperature in the dark, dehydrated through a graded ethanol series (50%, 70%, 95%, and 2 × 100%), and embedded in LX-112 resin (Ladd Research) using standard protocol (*67*). Sections (50 nm) cut parallel to the cellular monolayer were obtained using a Leica Ultracut 7 ultramicrotome, mounted on carbon/formvar-coated 300-mesh copper grids, and stained with uranyl acetate and lead citrate. Transmission electron microscopy (TEM) micrographs were obtained using a Helios CX-5 electron microscope equipped with a STEM3+ detector at 20 kV in high-resolution immersion mode and analyzed using FIJI software, with blinded analysis.

### Mitochondrial area measurements

Mitochondrial area was measured using a previously described protocol (*68*). In brief, HeLa cells were transfected with siRNA to deplete the target proteins, followed by fixation and immunofluorescence staining using a polyclonal rabbit anti-TOM20 antibody. To capture mitochondria distributed across different cellular planes, *z*-stacks spanning 3 μm in thickness were acquired for each cell, consisting of 15 serial images spaced 0.2 μm apart. Mitochondrial morphology was analyzed within a defined 225-μm^2^ region of interest (ROI) using ImageJ software [National Institutes of Health (NIH)]. Maximum intensity projections were generated from the *z*-stacks, and images were converted to 8-bit and thresholded using Otsu’s method. The “Analyze Particles” tool in ImageJ was then used to quantify the area of each mitochondrial fragment within the ROI.

### Protein expression and purification

Expi293F cells (2 × 10⁶; Life Technologies, A14527) were cultured in 1 liter of Expi293 expression medium (Life Technologies, A1435101) and transfected with 1 mg of DNA and 3 mg of sterile 25-kDa linear polyethyleneimine, prepared in Opti-MEM reduced-serum medium (Life Technologies, 31985070). The cells were incubated at 37°C with 8% CO_2_ and shaking at 125 rpm for 3 days, harvested by centrifugation at 300*g* for 15 min at 4°C, and washed with 1× PBS.

The S-Opa1 purification construct was expressed in *Escherichia coli* One Shot BL21 Star (DE3) cells (Life Technologies, C6010-03) grown in LB broth at 37°C. Protein expression was induced with 1 mM isopropyl-β-d-thiogalactoside at 18°C for 16 hours, when the culture reached an optical density at 600 nm of 0.6. Cells were harvested by centrifugation at 3000*g* for 15 min at 4°C. Both LACTB and S-Opa1 were subsequently purified using their C-terminal Strep II tags as described below.

All the subsequent steps were performed at 4°C or on ice. The cells were resuspended in 50 ml of lysis buffer [20 mM Hepes buffer (pH 7.4), 500 mM NaCl, 5 mM EDTA, 1 mM DTT, leupeptin (2 μg/ml), aprotinin (10 μg/ml), pepstatin A (2 μg/ml), calpeptin (1 μg/ml), calpain inhibitor I (1 μg/ml), 1 mM benzamidine, and 1:1000 dilution of universal nuclease; Thermo Fisher Scientific, 88702] per 5 ml of cell pellet. The cells were lysed using a high-pressure homogenizer (Microfluidics, M-110L), and 1% Triton X-100 was added after solubilization. Cell debris was removed by centrifugation at 40,000 rpm (185,000*g*) for 30 min using a Ti45 rotor (Beckman Coulter). The clarified supernatant was loaded onto Strep-Tactin Superflow resin (IBA, 2-1206-025) pre-equilibrated with lysis buffer, and washed with 30 column volumes wash buffer [10 mM Hepes (pH 7.4), 150 mM KCl, 1 mM EDTA, and 1 mM DTT]. The strep-tagged GFP fusion protein was eluted using strep elution buffer [10 mM Hepes (pH 7.4), 150 mM KCl, 1 mM DTT, and 2.5 mM desthiobiotin].

### Membrane nanotube assay

Lipid nanotubes were made as previously described (*44*, *45*), with all lipids sourced from Avanti Polar Lipids. Briefly, 2 μl of a 1 mM lipid stock (in chloroform) was deposited onto glass coverslips passivated with covalently attached polyethylene glycol, molecular weight 800 (Sigma-Aldrich, P2139). The coverslips were vacuum-dried and assembled into a flow cell (FCS2, Bioptechs). Lipids were hydrated by flowing 20 mM Hepes buffer (pH 7.4) containing 150 mM KCl. High flow rates of the same buffer facilitated the formation of an SLB at the lipid source and generated membrane nanotubes downstream. The flow was then halted, allowing the nanotubes to settle and adhere to defects on the glass surface. Reaction mixtures were introduced onto the nanotubes at flow rates 10 times slower than those used during nanotube preparation. The SLB formed during this process served as an in situ calibration standard for estimating nanotube dimensions (*44*, *45*). Experiments involving LACTB-GFP on the nanotubes were conducted at a total protein concentration of 1 μM in binding buffer [20 mM Hepes buffer (pH 7.4) containing 150 mM KCl and 1 mM MgCl_2_]. Temperature was maintained at 37°C using a temperature controller from Bioptechs.

### Fluorescence recovery after photobleaching

FRAP was performed on nanotubes following LACTB-GFP/6× His recruitment using a Dragonfly spinning-disk confocal microscope operated by Andor iQ3 software. Bleaching was achieved with a solid-state 405 smart diode laser (100 mW) set at 40% output power, delivering a 500-ms exposure to a fixed area of 2 μm^2^. Images were captured in both the 488-nm (protein) and 561-nm (membrane) channels before bleaching and 1 s postbleach. To minimize photobleaching during acquisition, imaging parameters were standardized, and the mean postbleach intensity was normalized to the mean prebleach intensity.

### LACTB binding assay

LACTB-GFP (1 μM) was incubated with nanotubes of the respective membrane composition for 10 min, followed by washing with 400 μl of binding buffer. The nanotubes were subsequently imaged. Protein density was calculated by dividing the mean protein intensity by the mean membrane intensity and multiplying by 100, with the area held constant for both nanotubes and SLB.

### Image and statistical analysis

All graphical representations and statistical analyses were performed using GraphPad Prism (version 10.4.1). Statistical significance was determined using one-way analysis of variance (ANOVA) or the Mann-Whitney test, as appropriate. The number of independent experiments, number of cells or mitochondria analyzed, and the statistical tests performed are provided in the respective figure legends. Image analysis was conducted using ImageJ Fiji (version 2.14.0/1.54f, NIH). TEM micrographs were manually analyzed using ImageJ after blinding. Mitochondria exhibiting swollen morphology and expanded cristae were classified as aberrant. Mitochondrial width was measured by drawing a line across the outer mitochondrial membrane from one side to the other, while cristae density was determined by counting the number of cristae within a mitochondrion and dividing by total mitochondrial length (in micrometer). The number of experimental replicates for each assay is specified in the corresponding figure legends.

## Supplementary Materials

### The PDF file includes:

Figs. S1 to S15 Table S1 Legend for table S2 Legends for movies S1 to S7 Data S1 (Uncropped Western blots)
