# Supplementary material for: The tumor suppressor LACTB remodels mitochondria to promote cytochrome c release and apoptosis
Source: Sci Adv. 2025 Nov 12;11(46):eadx7809. doi: 10.1126/sciadv.adx7809 (PMC12609066; doi:10.1126/sciadv.adx7809)

Science Advances
AAAS

Supplementary Materials for

The tumor suppressor LACTB remodels mitochondria to promote cytochrome c release and apoptosis

Sukrut C. Kamerkar *et al.*

Corresponding author: Henry N. Higgs, henry.higgs@dartmouth.edu

Sci. Adv. **11**, eadx7809 (2025)
DOI: 10.1126/sciadv.adx7809

The PDF file includes:

Figs. S1 to S15
Table S1
Legend for table S2
Legends for movies S1 to S7
Data S1 (Uncropped Western blots)

Other Supplementary Material for this manuscript includes the following:

Table S2
Movies S1 to S7

Fig. S1.

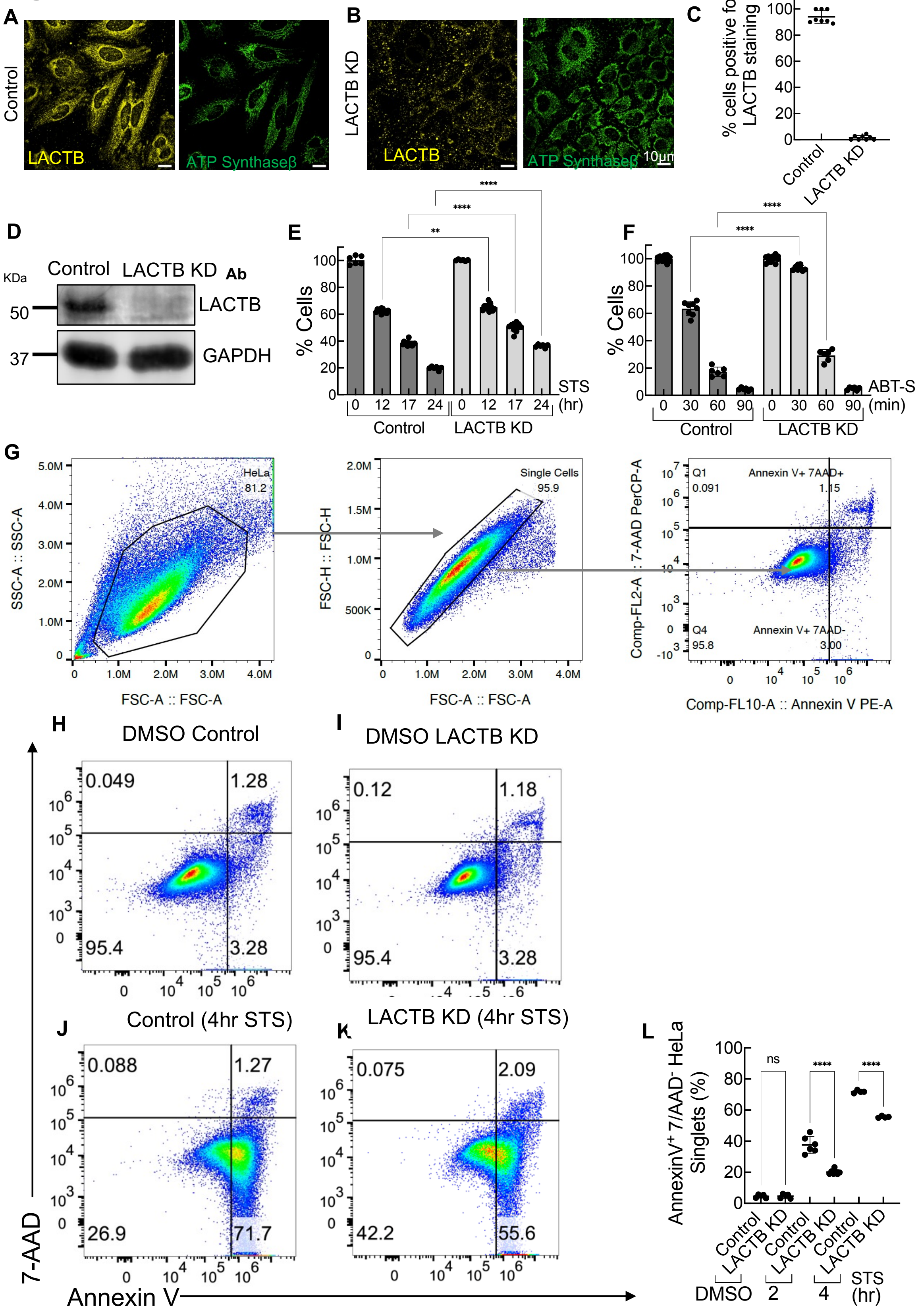
A
Control
LACTB
ATP Synthaseβ
B
LACTB KD
LACTB
ATP Synthaseβ
10μm
C
% cells positive for LACTB staining
Control
LACTB KD
D
KDa
Control
LACTB KD
Ab
LACTB
GAPDH
50
37
E
% Cells
STS (hr)
Control
LACTB KD
F
% Cells
ABT-S (min)
Control
LACTB KD
G
HeLa 81.2
SSC-A :: SSC-A
FSC-A :: FSC-A
Single Cells 95.9
FSC-H :: FSC-H
Q1 0.091
Annexin V+ 7AAD+ 1.15
Q4 95.8
Annexin V+ 7AAD- 3.00
Comp-FL2-A :: 7-AAD PerCP-A
Comp-FL10-A :: Annexin V PE-A
H
DMSO Control
0.049
1.28
95.4
3.28
I
DMSO LACTB KD
0.12
1.18
95.4
3.28
J
Control (4hr STS)
0.088
1.27
26.9
71.7
K
LACTB KD (4hr STS)
0.075
2.09
42.2
55.6
7-AAD
Annexin V
L
AnnexinV+ 7/AAD- HeLa Singlets (%)
ns
Control
LACTB KD
DMSO
2
4
STS (hr)

Figure S1. LACTB knockdown reduces apoptosis

(A–C) Immunofluorescence staining of endogenous LACTB and ATP Synthase β in control (A) and LACTB knockdown (B) HeLa cells. Quantification of the number of cells (C) showing detectable mitochondrial LACTB staining is shown. N ≥ 88 cells; N = 1 independent experiment.

(**D**) Western blot for LACTB in control or LACTB knockdown (KD) B16-F10 cells. GAPDH used as loading control.

(**E**) Quantification of SRB assay in control and LACTB KD B16-F10 cells treated with 1 μM staurosporine (STS) for various times. N = 2.

(**F**) Quantification of SRB assay in control and LACTB KD B16-F10 cells treated with ABT-S for various times. N = 2.

(**G**) Representative gating strategy used for flow cytometry analysis of HeLa cells for AnnexinV and 7-AAD staining. Cells were initially gated based on forward scatter area (FSC-A) versus side scatter area (SSC-A) to identify the population of interest, followed by FSC-A versus forward scatter height (FSC-H) to exclude doublets.

(**H**, **I**) Annexin-V and 7-AAD staining of control (H) and LACTB KD (I) HeLa cells treated with DMSO for 4 hours..

(**J**, **K**) Annexin-V and 7-AAD staining of control (J) and LACTB KD (K) HeLa cells treated with 1 μM STS for 4 hours.

(**L**) Quantification of Annexin-V^{+}/7^{-}AAD -HeLa singlets from three independent control and LACTB KD experiments upon STS treatment.

****p<0.0001, **p<0.01 (one-way ANOVA). Data are presented as mean ± SD. N represents the number of independent experiments.

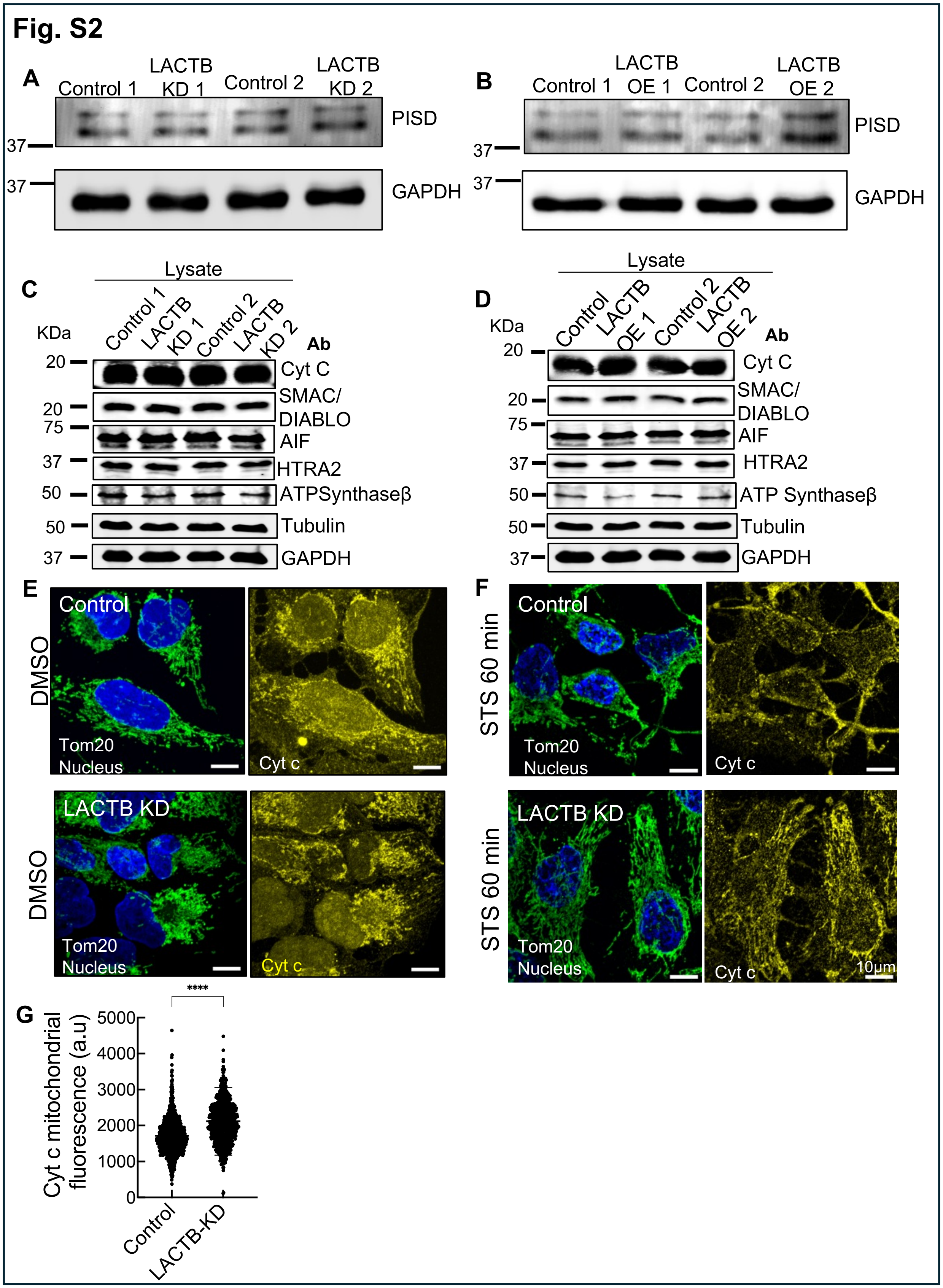
Fig. S2
A
Control 1
LACTB KD 1
Control 2
LACTB KD 2
PISD
GAPDH
37
B
Control 1
LACTB OE 1
Control 2
LACTB OE 2
PISD
GAPDH
C
Lysate
KDa
Ab
Cyt C
SMAC/ DIABLO
AIF
HTRA2
ATPSynthaseβ
Tubulin
GAPDH
D
ATP Synthaseβ
E
DMSO
Control
LACTB KD
Tom20
Nucleus
Cyt c
F
STS 60 min
10µm
G
Cyt c mitochondrial fluorescence (a.u)
****
Control
LACTB-KD

Figure S2: LACTB knockdown and overexpression do not alter expression levels of mitochondrially-released factors or PISD

(**A,B**) PISD expression levels in control and LACTB KD (A; two independent knockdowns) or control and LACTB OE HeLa cells (B; two independent overexpression lines).

(**C,D**) Western blot analysis of total cell extracts from control and LACTB KD (C) or control and LACTB OE (D) HeLa cells for cytochrome c and other mitochondrial proteins. GAPDH and tubulin used as loading controls. Experiments were performed using two independent LACTB KD samples or LACTB OE lines.

(**E,F**) Immunofluorescence staining for cytochrome c (yellow) and Tom20 (green) in U2-OS cells under DMSO treatment (E) or 1 μM STS for 1 hour (F). Blue, DAPI.

(**G**) Quantification of mitochondrial cytochrome c levels upon 1-hour STS treatment in U2-OS cells ($N_{mito} \geq 735$, $N_{cells} = 15$) ****$p < 0.0001$ (Mann–Whitney test). Data are presented as mean ± SD.

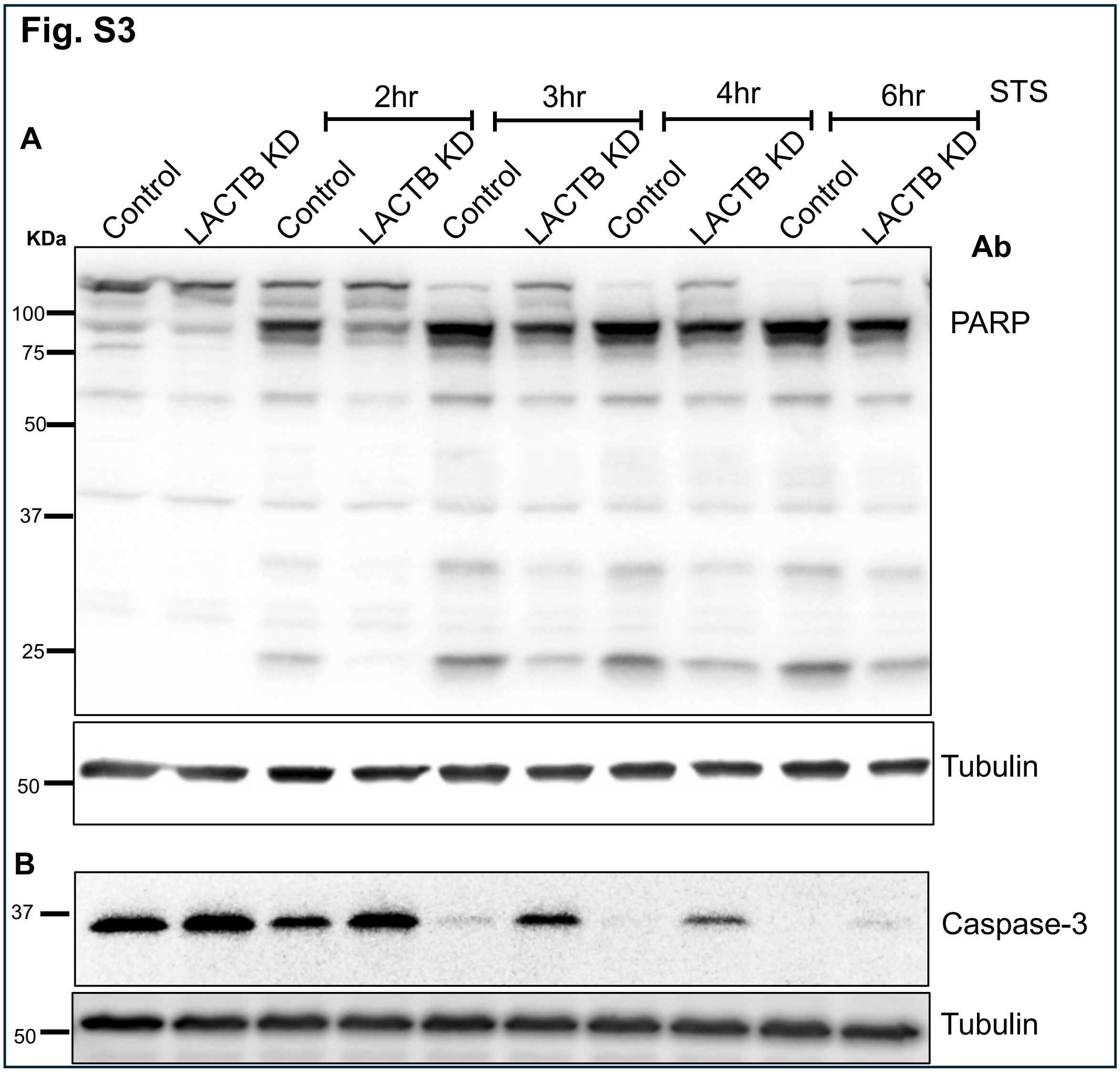

Figure S3. LACTB KD delays PARP cleavage and caspase-3 activation during apoptosis

(**A**) Western blot analysis of PARP cleavage in total cell extracts from control and LACTB KD HeLa cells treated with DMSO (1 hour) or staurosporine (STS; 1 µM) for the indicated time intervals. Tubulin was used as a loading control.

(**B**) Western blot showing total caspase-3 levels in total cell extracts from control and LACTB KD HeLa cells under the same conditions as in (A). Tubulin was used as a loading control.

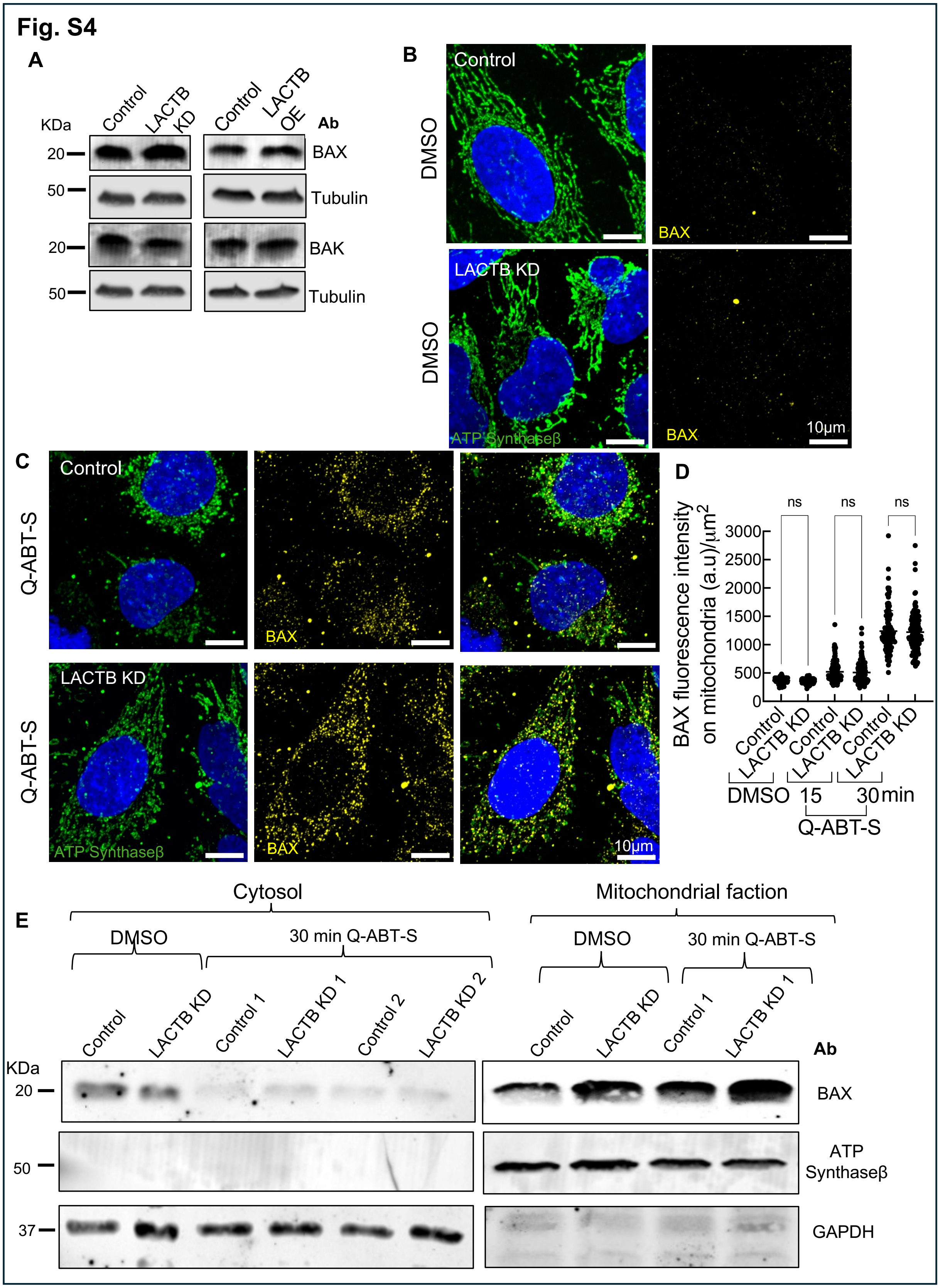
Fig. S4
A
KDa
Control
LACTB KD
Control
LACTB OE
Ab
20
50
20
50
BAX
Tubulin
BAK
Tubulin
B
Control
DMSO
BAX
LACTB KD
DMSO
ATP Synthaseβ
BAX
10μm
C
Control
Q-ABT-S
BAX
LACTB KD
Q-ABT-S
ATP Synthaseβ
BAX
10μm
D
BAX fluorescence intensity on mitochondria (a.u)/μm2
ns
ns
ns
3000
2500
2000
1500
1000
500
0
Control
LACTB KD
Control
LACTB KD
Control
LACTB KD
DMSO
15
30min
Q-ABT-S
E
Cytosol
Mitochondrial faction
DMSO
30 min Q-ABT-S
DMSO
30 min Q-ABT-S
Control
LACTB KD
Control 1
LACTB KD 1
Control 2
LACTB KD 2
Control
LACTB KD
Control 1
LACTB KD 1
Ab
KDa
20
50
37
BAX
ATP Synthaseβ
GAPDH

Figure S4: LACTB does not influence BAX recruitment to mitochondria

(**A**) Western blot analysis of BAX and BAK levels in total cell extracts from control, LACTB KD or LACTB OE HeLa cells. Tubulin used as a loading control.

(**B**, **C**) Immunofluorescence staining of endogenous BAX (yellow) in control and LACTB KD HeLa cells treated with DMSO (B) or ABT-S (C) for 30 min. Prior to ABT-S treatment, the pan-caspase inhibitor Q-VD-OPh (20 μM) was added for 1-hour. ATP synthase β staining for mitochondria in green, and DAPI staining for nuclei in blue. BAX signal intensity was brightness and contrast adjusted to specifically highlight its mitochondrial localization.

(**D**) Quantification of mitochondrial BAX fluorescence intensity (intensity per μm^2 mitochondrial area) in control and LACTB KD HeLa cells treated with Q-ABT-S for 15 or 30 minutes. Data represent mean ± SD from 2 independent experiments, with $N_{cells} \geq 104$. Statistical analysis using one-way ANOVA indicates no significant difference (ns).

(**E**) Western blot analysis of BAX, GAPDH, and ATP synthase in cytosolic and mitochondrial extracts from control and LACTB KD HeLa cells after 30 min DMSO or Q-ABT-S treatment. Experiment for cytosolic extract conducted with two independent LACTB KD samples.

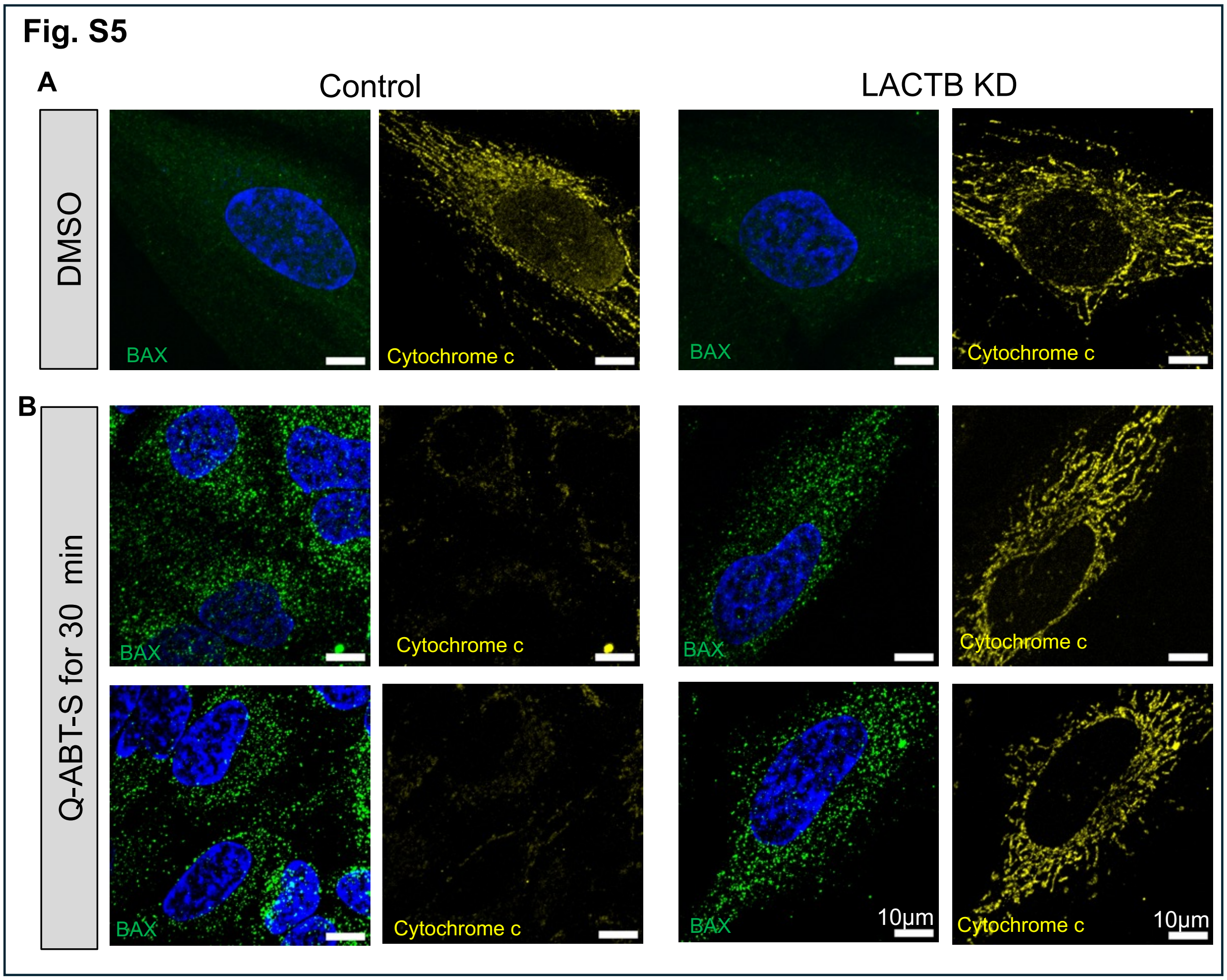

Figure S5. LACTB influences cytochrome c release from mitochondria during apoptosis

(**A**) Immunofluorescence staining of endogenous BAX (green) in control and LACTB KD HeLa cells treated with DMSO. Cytochrome c is shown in yellow (mitochondria), and nuclei are stained with DAPI (blue).

(**B**) Immunofluorescence staining of endogenous BAX (green) in control and LACTB KD HeLa cells treated for 30 minutes with ABT-S (10 µM ABT-737 and 2 µM S63845) in the presence of the pan-caspase inhibitor Q-VD-OPh (20 µM, preincubated for 1 hour). Cytochrome c is shown in yellow (mitochondria), and nuclei are stained with DAPI (blue).

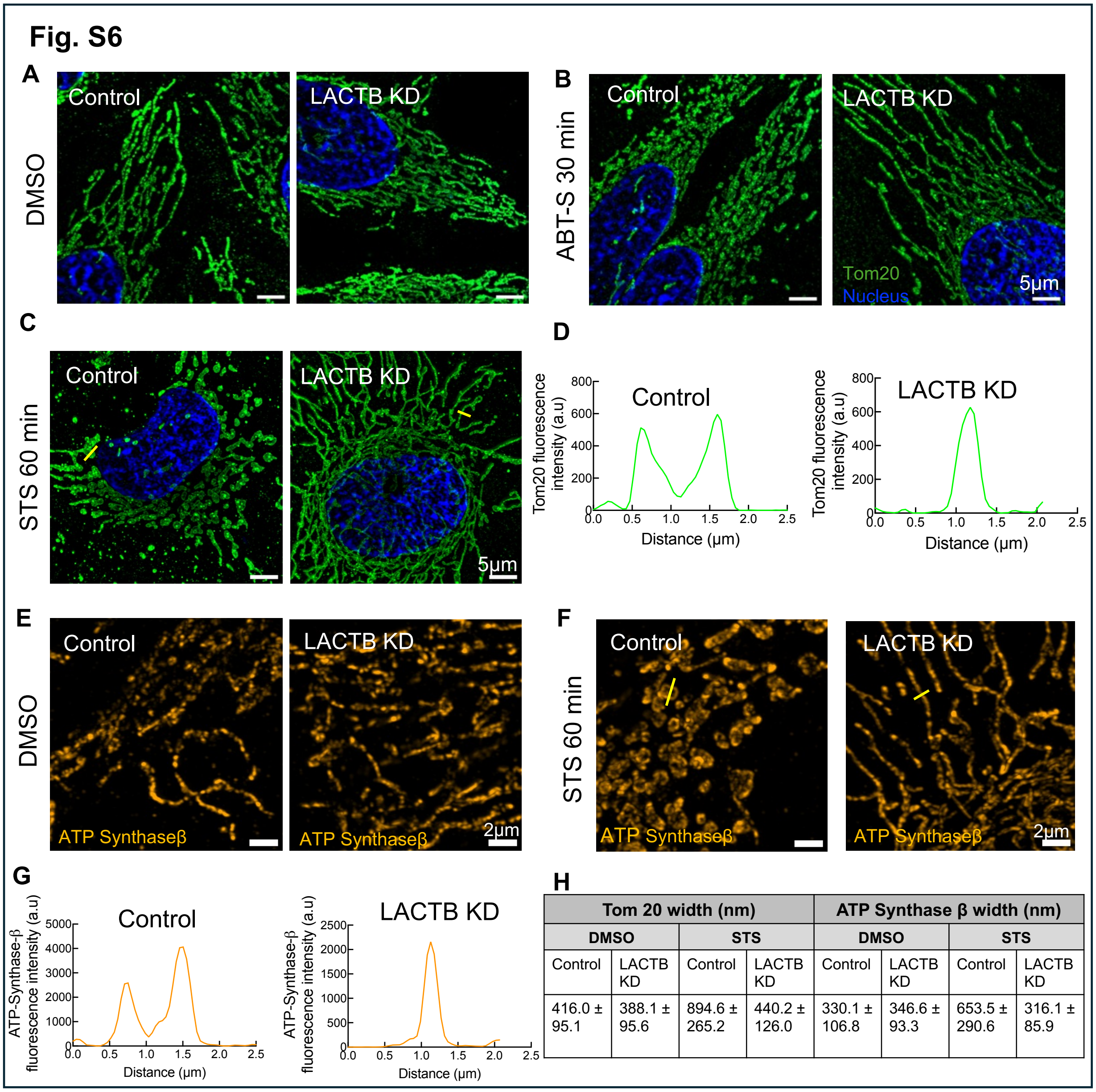

Tom 20 width (nm)				ATP Synthase β width (nm)			
DMSO		STS		DMSO		STS	
Control	LACTB KD	Control	LACTB KD	Control	LACTB KD	Control	LACTB KD
416.0 ± 95.1	388.1 ± 95.6	894.6 ± 265.2	440.2 ± 126.0	330.1 ± 106.8	346.6 ± 93.3	653.5 ± 290.6	316.1 ± 85.9

Figure S6: LACTB mediates mitochondrial remodelling upon apoptosis induction

(**A-C**) Full images of magnified insets from Fig 3A-C showing immunofluorescence staining of Tom20 (green) in control and LACTB KD HeLa cells treated with 1 hr DMSO (A), 30 min ABT-S (B) or 1 hr STS (C).

(**D**) Line profile analysis of Tom20 fluorescence intensity in control and LACTB KD HeLa cells treated with STS for 1 hour. Lines shown in panel C..

(**E,F**) Immunofluorescence staining of ATP Synthase β (orange) in control or LACTB KD HeLa cells upon DMSO (E) or staurosporine (STS) treatment (F) for 60 min.

(**G**) Line scan analysis of ATP Synthase β fluorescence intensity in control and LACTB KD

HeLa cells treated with STS for 60 min.

(**H**) Table summarizing mitochondrial widths (mean ± SD, in nm), as measured by Tom20 or ATP synthase b markers, of control or LACTB KD HeLa cells upon DMSO or STS treatment.

Fig. S7

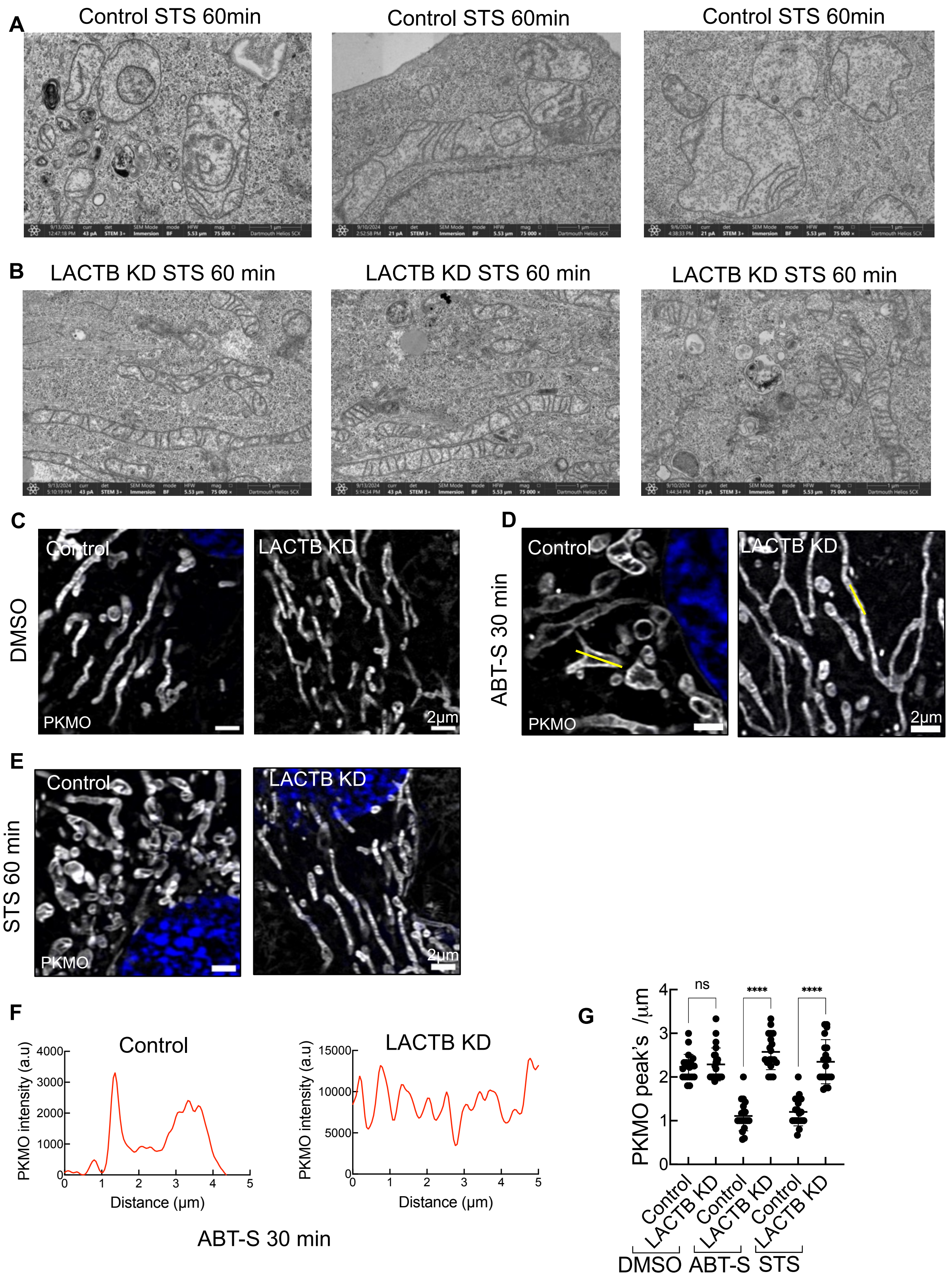

A
Control STS 60min
Control STS 60min
Control STS 60min
B
LACTB KD STS 60 min
LACTB KD STS 60 min
LACTB KD STS 60 min
C
DMSO
Control
LACTB KD
PKMO
2μm
D
ABT-S 30 min
Control
LACTB KD
PKMO
2μm
E
STS 60 min
Control
LACTB KD
PKMO
2μm
F
Control
PKMO intensity (a.u)
Distance (μm)
LACTB KD
PKMO intensity (a.u)
Distance (μm)
ABT-S 30 min
G
PKMO peak's /μm
ns
****
****
Control
LACTB KD
Control
LACTB KD
Control
LACTB KD
DMSO
ABT-S
STS

Figure S7. LACTB KD preserves IMM morphology during apoptosis

(**A**, **B**) TEM micrographs of mitochondria in control (E) and LACTB KD (F) HeLa cells treated with STS for 60 min

(**C**-**E**) PKmito Orange FX (PKMO) staining (cristae marker, in grey) in control siRNA and LACTB KD HeLa cells upon DMSO (C), 30 min ABT-S (D), or 1-hr STS (E) treatment. Blue, DAPI.

(**F**) Line scan analysis of PKMO fluorescence intensity in control and LACTB KD cells treated with ABT-S for 30 min.

(**G**) Quantification of PKMO intensity peaks (per micron) using line profile analysis of mitochondria. $N_{mito} \geq 19$. Error bars represent mean ± SD. ****$p < 0.0001$; ns, not significant (one-way ANOVA).

Fig. S8

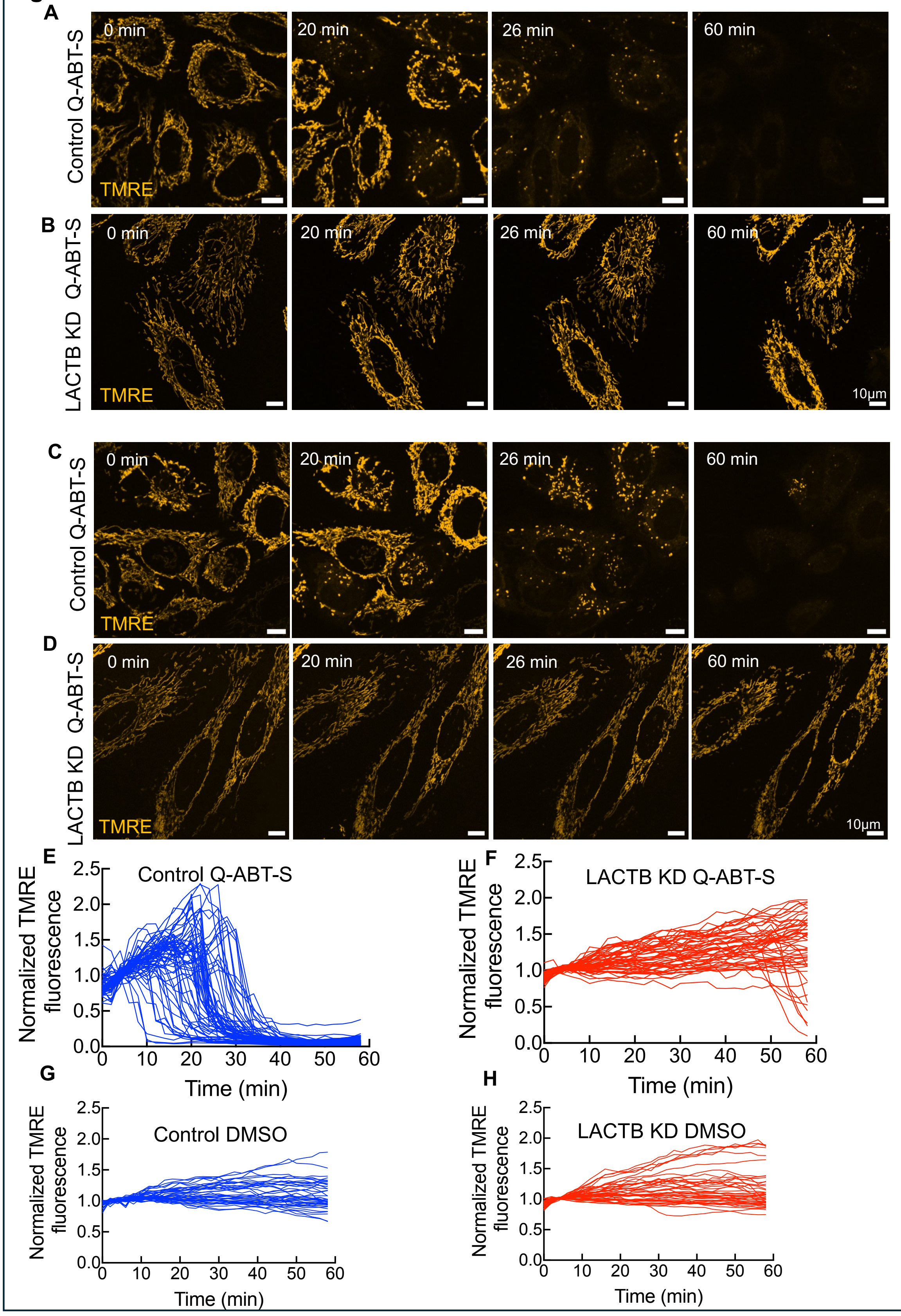
A
B
C
D
E
F
G
H
Control Q-ABT-S
LACTB KD Q-ABT-S
0 min
20 min
26 min
60 min
TMRE
10µm
Normalized TMRE fluorescence
Time (min)
Control DMSO
LACTB KD DMSO

Figure S8. LACTB KD inhibits mitochondrial depolarization upon apoptotic stimulation

(**A**–**D**) Live-cell imaging of mitochondrial depolarization using TMRE (orange) in control (A, C) and LACTB KD (B, D) HeLa cells treated with ABT-S (10 µM ABT-737 and 2 µM S63845) in the presence of the pan-caspase inhibitor Q-VD-OPh (20 µM, 1-hour preincubation).

(**E**) Quantification of mitochondrial membrane depolarization kinetics in control siRNA-treated cells following Q-ABT-S treatment. N = 2 independent experiments; N_{cells} = 65.

(**F**) Quantification of mitochondrial membrane depolarization kinetics in LACTB KD cells following Q-ABT-S treatment. N = 2 independent experiments; N_{cells} = 55.

(**G**, **H**) Quantification of mitochondrial membrane depolarization kinetics in control (G) and LACTB KD (H) cells under DMSO treatment. N = 1 independent experiment; N_{cells} = 39 for control and 43 for LACTB KD.

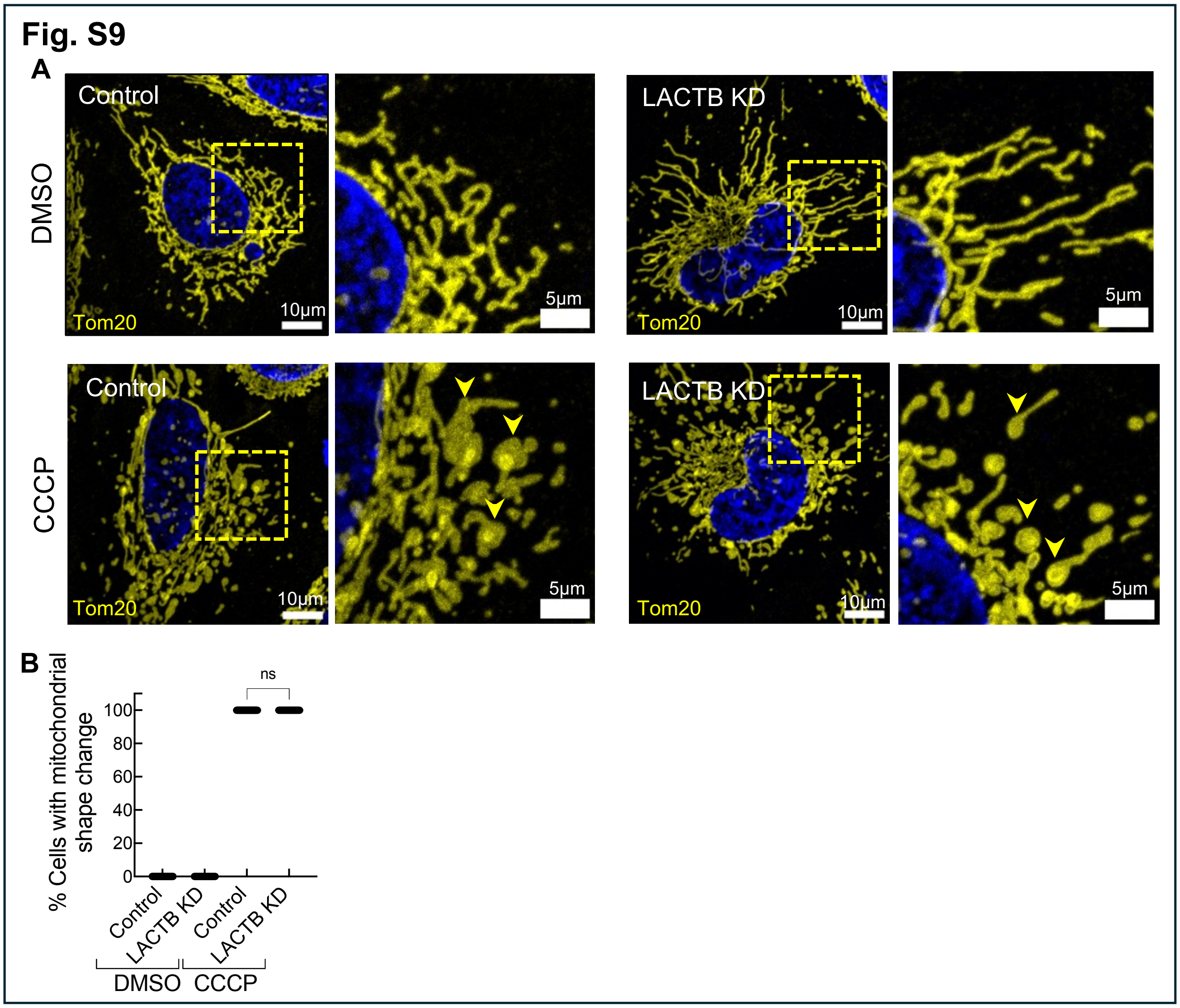

Figure S9: LACTB knockdown does not affect mitochondrial remodeling induced by CCCP treatment.

(**A**) Immunofluorescence staining of Tom20 (yellow) in control siRNA and LACTB KD HeLa cells after 20 min DMSO or CCCP (20 µM) treatment. Blue, DAPI. Panels to right are zooms of boxed regions.

(**B**) Scatter plot showing blinded analysis of cells undergoing mitochondrial shape change (assessed by Tom20 immunofluorescence) in control siRNA and LACTB KD HeLa cells after 20 min of DMSO or CCCP (20 µM) treatment. $N_{cells} \geq 24$. Error bars represent mean ± SD. ****$p < 0.0001$; ns, not significant (one-way ANOVA).

Fig. S10

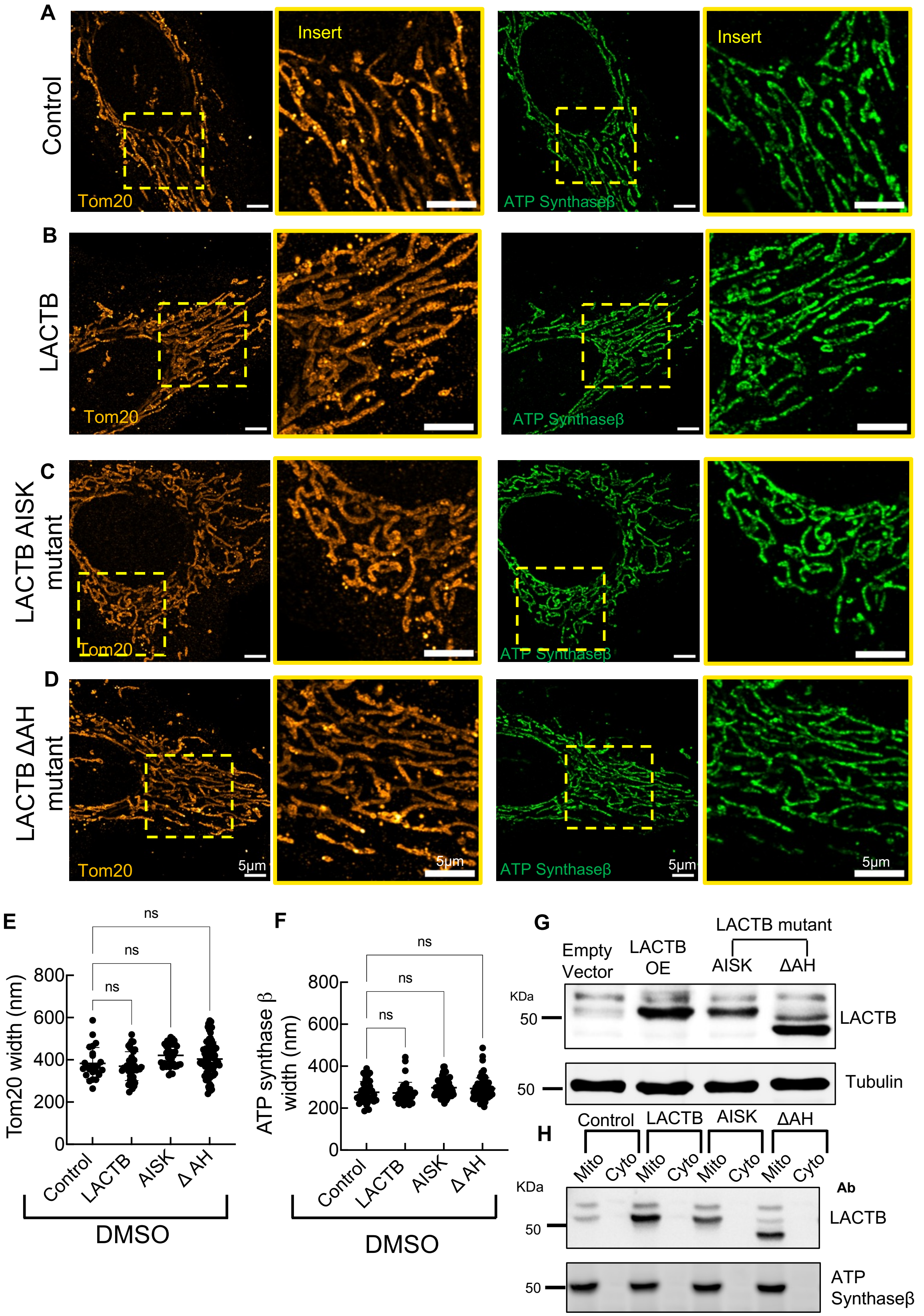
A
Control
Insert
Tom20
ATP Synthaseβ
B
LACTB
C
LACTB AISK mutant
D
LACTB ΔAH mutant
5µm
E
Tom20 width (nm)
ns
Control
LACTB
AISK
Δ AH
DMSO
F
ATP synthase β width (nm)
G
Empty Vector
LACTB OE
LACTB mutant
AISK
ΔAH
KDa
50
LACTB
Tubulin
H
Control
Mito
Cyto
Ab
ATP Synthaseβ

145

Figure S10. LACTB and its mutants do not alter mitochondrial width under basal conditions

(**A–D**) Immunofluorescence staining of Tom20 (red) and ATP Synthase β (green) in control (A), LACTB-overexpressing (B), LACTB active-site mutant (AISK) (C), and LACTB amphipathic helix mutant (ΔAH; putative membrane-binding mutant) (D) cells treated with DMSO for 20 minutes.

(**E-F**) Quantification of mitochondrial width based on Tom20 and ATP Synthase β fluorescence under ABT-S treatment. Data represent N = 1 independent experiments; $N_{mito} \geq 27$. Error bars indicate mean ± SD. ns, not significant (one-way ANOVA).

(**G**) Western blot confirming stable overexpression of LACTB and its mutants in HeLa cells using the piggyBac transposon system.

(**H**) Mitochondrial fractions were isolated from HeLa cells stably expressing wild-type LACTB or its mutants. Western blotting was performed to confirm mitochondrial enrichment of LACTB and its mutants.

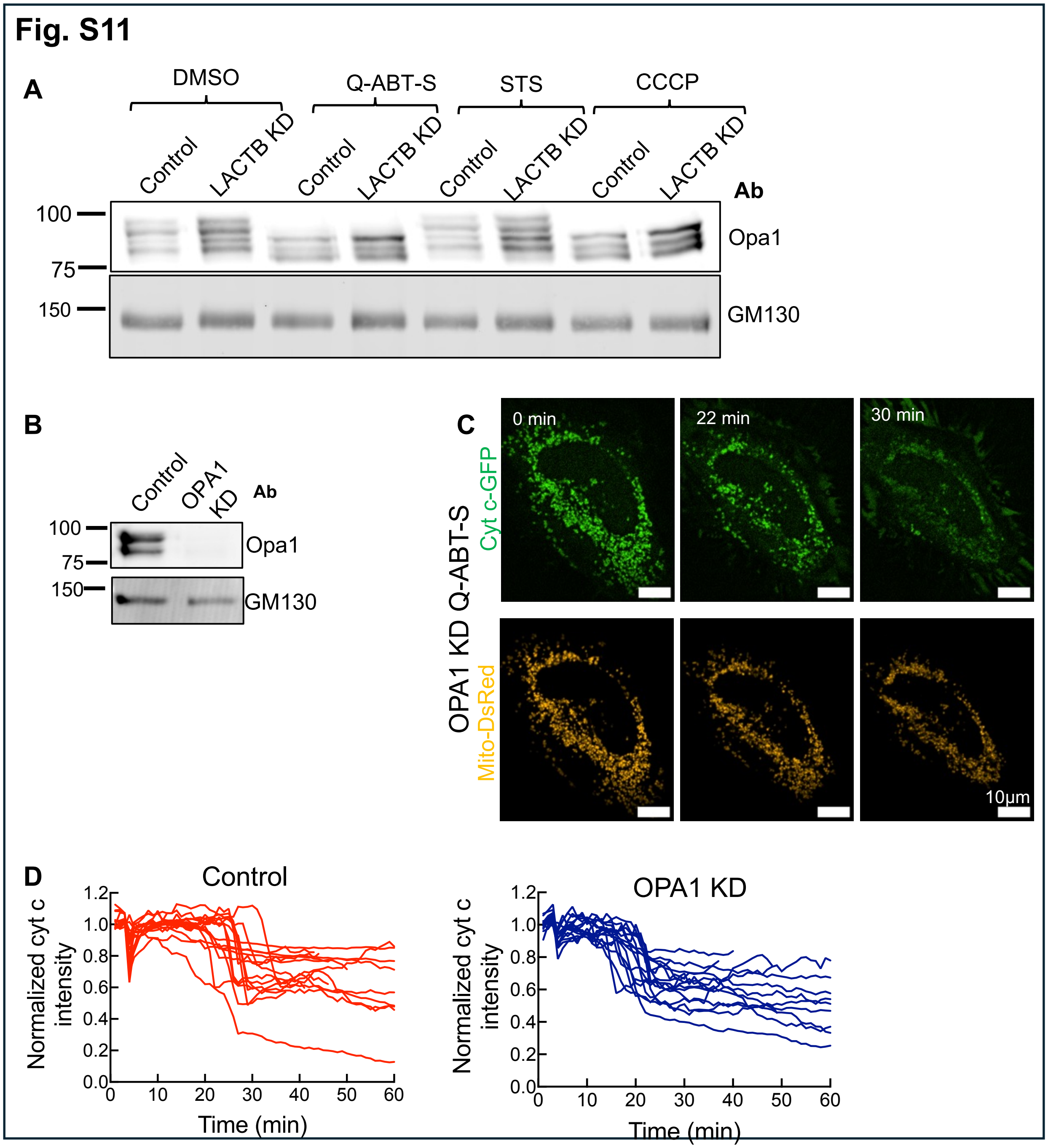

Figure S11: LACTB action is independent of OPA1

(**A**) Western blot assessing OPA1 processing in control and LACTB KD HeLa cells under DMSO (1hour), Q-ABT-S (30min), STS (1hour), and CCCP (20 min) treatments. GM130, loading control.

(**B**) Western blot of OPA1 in control and OPA1 KD HeLa cells. GM130, loading control.

(**C**) Live-cell imaging of GFP-tagged cytochrome c release in OPA1 KD HeLa cells treated with

Q-ABT-S for the indicated times.

(**D**) Quantification of cytochrome c release kinetics after Q-ABT-S treatment in control and OPA1 KD HeLa cells. N = 3 independent experiments; N_{cells}= 16.

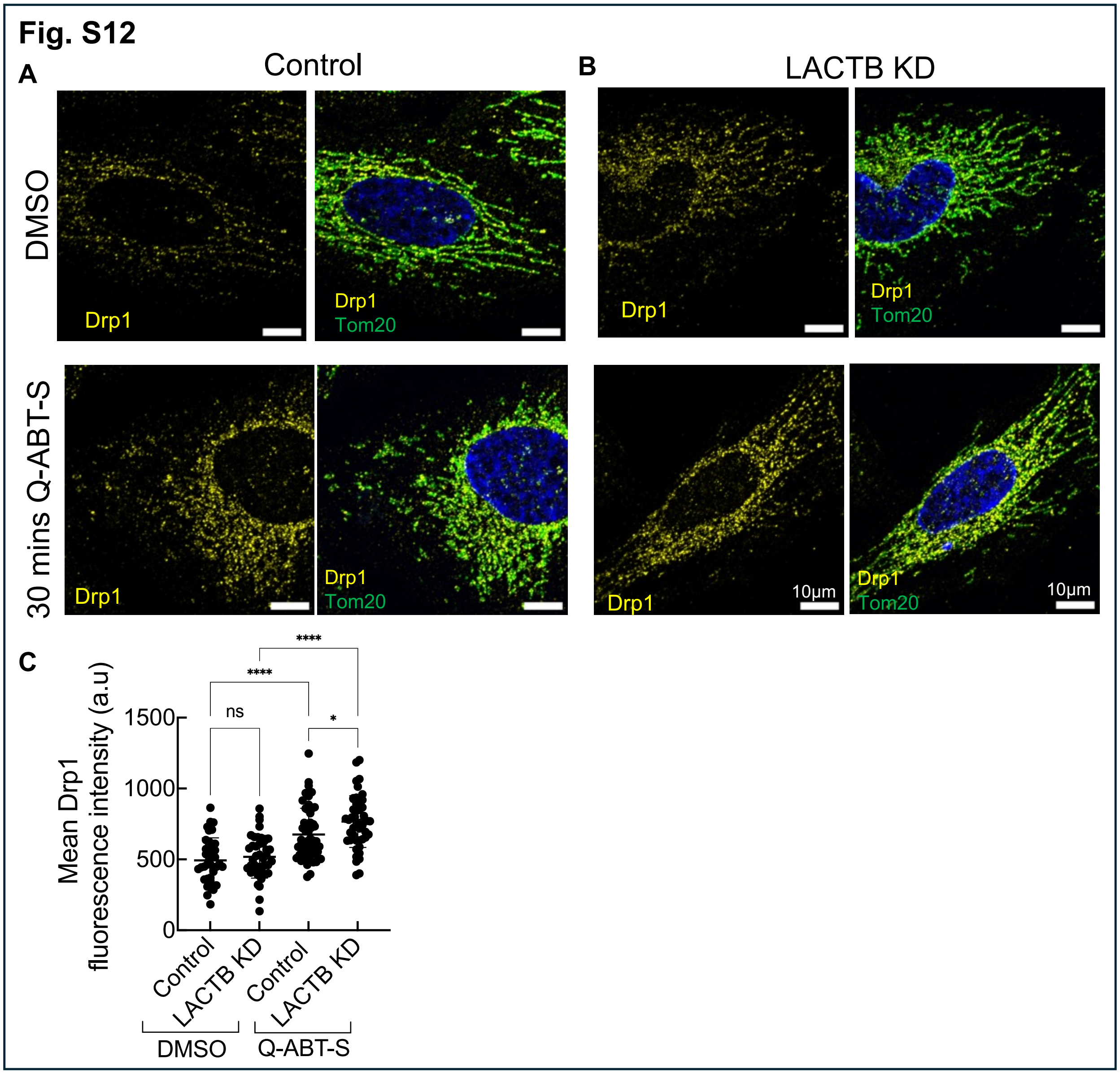

Figure S12. LACTB KD does not alter Drp1 recruitment to mitochondria during apoptosis induction.

(**A**) Immunofluorescence staining of Drp1 (yellow) and Tom20 (green) in control HeLa cells treated with DMSO or ABT-S (10 μM ABT-737 and 2 μM S63845 for 30 minutes) in the presence of the pan-caspase inhibitor Q-VD-OPh (20 μM, 1-hour preincubation).

(**B**) Immunofluorescence staining of Drp1 (yellow) and Tom20 (green) in LACTB knockdown HeLa cells under the same conditions as in (A).

(**C**) Quantification of mean Drp1 intensity on mitochondria in control and LACTB knockdown

cells under DMSO and Q-ABT-S treatment. Data represent N = 2 independent experiments; $N_{cells} \geq 39$. Error bars indicate mean ± SD. ****$p < 0.0001$ (one-way ANOVA).

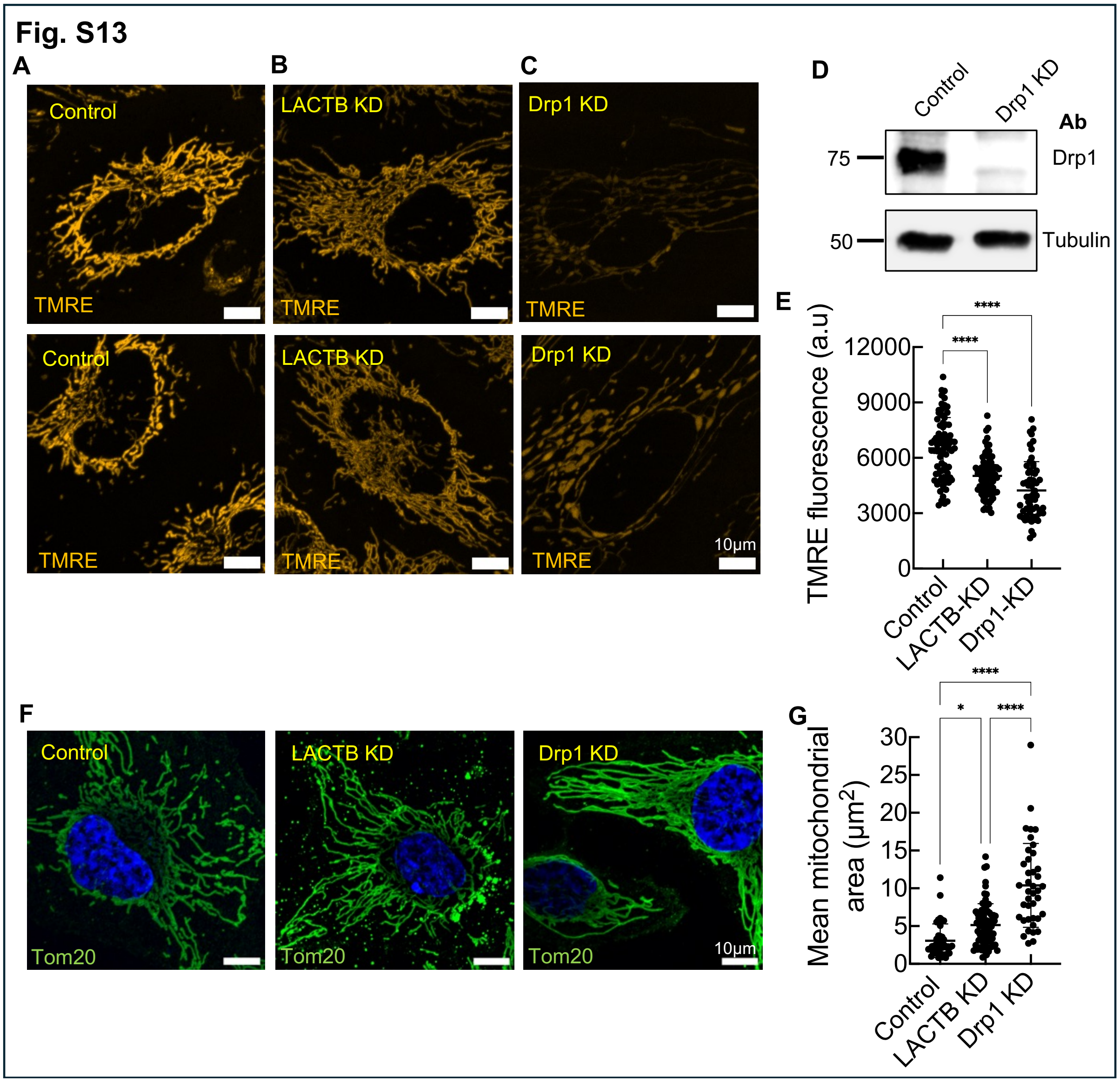

Figure S13. Effect of LATCB KD on mitochondrial morphology and potential

(**A–D**) Staining of mitochondrial membrane potential in control (A), LACTB KD (B), and Drp1 KD (C, D) HeLa cells.

(**E**) Quantification of mitochondrial membrane potential. Data represent N = 2 independent experiments; $N_{cells} \geq 57$. Error bars indicate mean ± SD. ****$p < 0.0001$

(**F–G**) Immunofluorescence staining of Tom20 (green) in control, LACTB knockdown, and Drp1 knockdown HeLa cells (F), and quantification of mitochondrial area (G). Data represent N = 2 independent experiments; $N_{cells} \geq 41$. Error bars indicate mean ± SD. ****$p < 0.0001$; (one-way ANOVA).

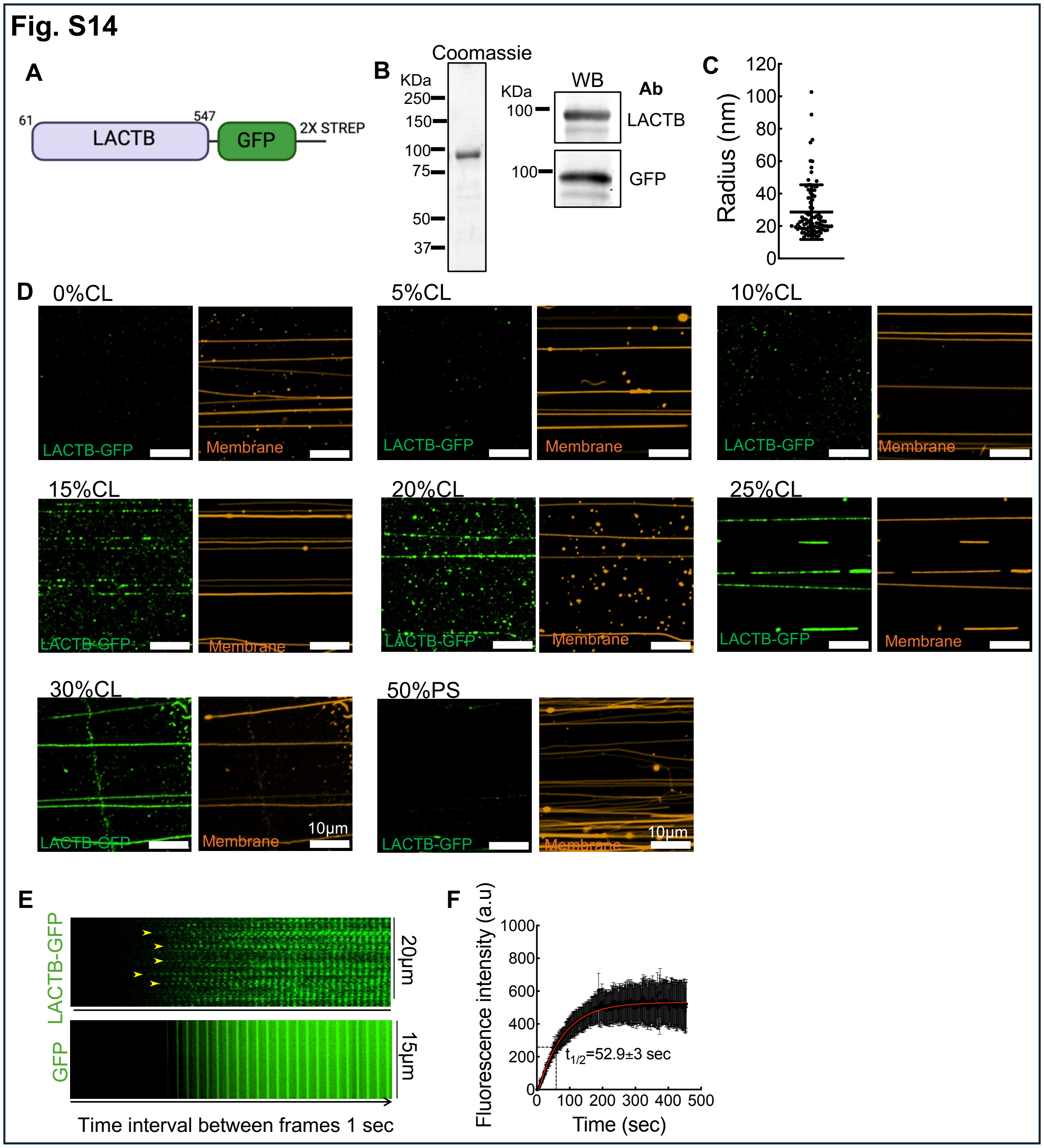

Figure S14: Characterization of membrane binding by LACTB

(**A**) Cartoon representation of the LACTB construct used for biochemical studies. The N-terminal 60 amino acids are LACTB's mitochondrial targeting sequence, and were removed from the construct. 2x STREP = tandem Strep purification tag.

(**B**) Coomassie-stained SDS-PAGE gel and western blot of purified LACTB-GFP.

(**C**) Distribution of radii for membrane nanotubes in the system used here, computed using a previously described method(*44*, *45*). Error bars represent mean ± SD. N = 3 experiments;

nanotubes = 90

(**D**) Representative micrographs of LACTB-GFP binding to membrane nanotubes (10 min incubation) as a function of cardiolipin concentration. Lipid mixtures contain PC and 0.5 mol% RhPE. Varying % of CL or phosphatidylserine (PS) are added (with corresponding % of PC removed).

(**E**) Representative timelapse montage showing nucleation (yellow arrowheads) and growth of LACTB-GFP scaffolds on nanotubes. Control experiments with uniform recruitment of 6His-GFP (onto nanotubes containing Ni-NTA lipid) were also performed. Lipid compositions: DOPC:CL:RhPE (74.5:25:0.5 mol%) for LACTB-GFP; DOPC:PS:DGS-NiNTA:RhPE (79.5:15:5:0.5 mol%) for 6His-GFP.

(**F**) Rate of LACTB-GFP binding to nanotubes. N = 2 experiments; n_{tubes} =10

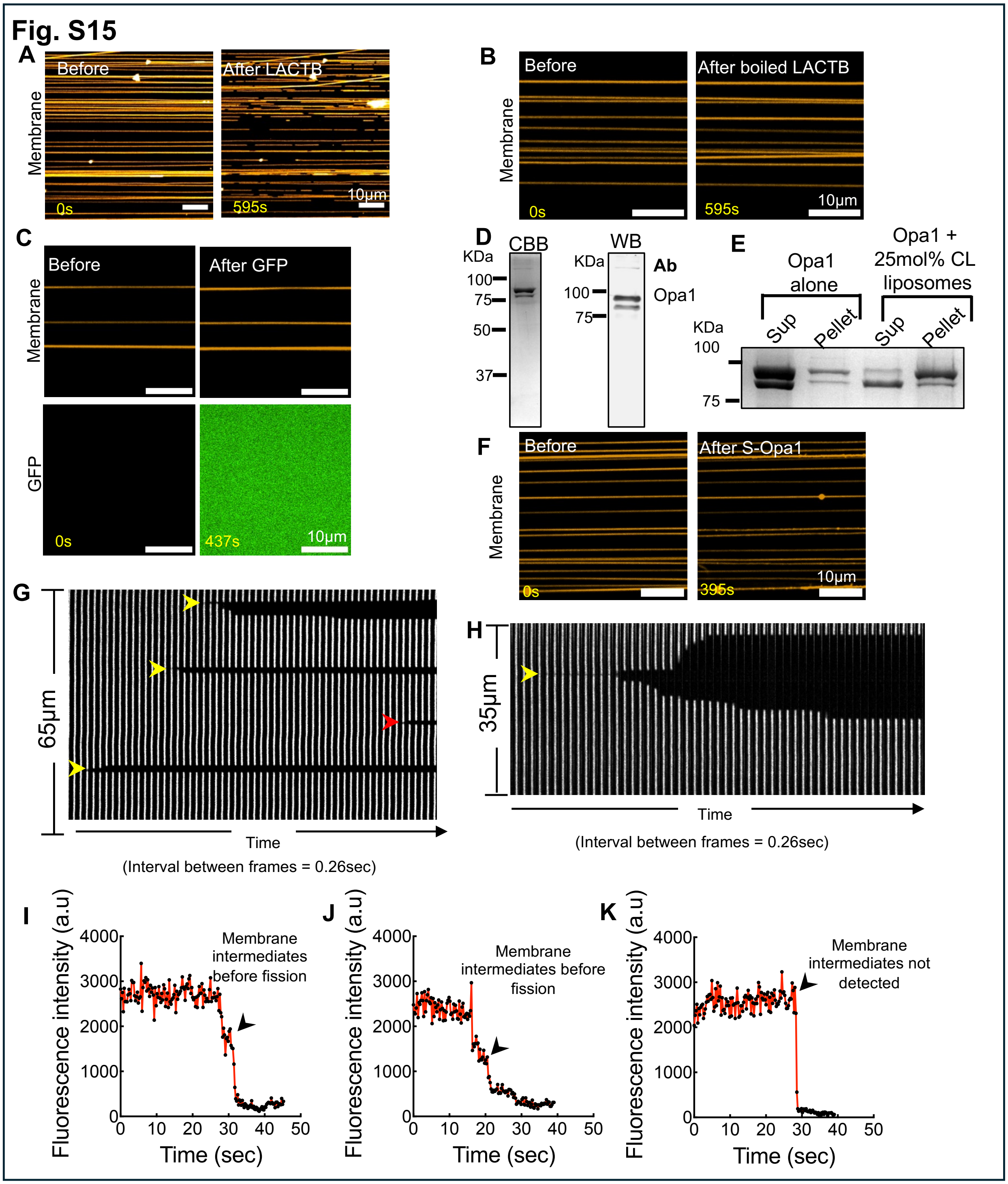

Figure S15: LACTB-mediated membrane fission

(**A**) Full imaging field from which the inset in Figure 5i was taken, before and 10 min after flowing LACTB-GFP onto nanotubes containing 25 mol% cardiolipin.

(**B**) Representative images showing membrane nanotubes containing 25 mol% cardiolipin before

and after flowing in boiled LACTB-GFP (10min incubation).

(**C**) Representative images showing membrane nanotubes containing 25 mol% cardiolipin before and after flowing 1 μM GFP.

(**D**) Coomassie-stained SDS-PAGE gel and western blot of purified S-Opa1.

(**E**) S-Opa1 was incubated with liposomes containing 25 mol% cardiolipin, followed by ultracentrifugation to separate the supernatant (Sup) and pellet (Pellet) fractions. Coomassie staining was used to assess S-Opa1 association with liposomes.

(**F**) Representative images showing membrane nanotubes containing 25 mol% cardiolipin before and after flowing 1 μM S-Opa1 in presence of 1mM GTP and 1mM $MgCl_2$.

(**G-H**) Representative kymographs of nanotubes during LACTB-mediated remodelling, showing fission events (arrowheads). Constriction is marked by a decrease in fluorescence intensity prior to fission (indicated by yellow arrowheads). A fission event without detectable prior constriction is also shown (red arrowhead).

(**I-K**) Single-pixel fluorescence traces extracted from kymographs highlighting the kinetics of membrane nanotube constriction before fission events. Panel (I) and (J) show two examples of constriction before fission, while panel (K) shows a fission event without detectable prior constriction. Fission event marked by black arrow head.

Table S1.

Details of antibodies used in this study, including sources, catalog numbers, and dilutions.

Antibody	Source	Catalog number	Dilution
Rabbit anti-LACTB	Proteintech	18195-1-AP	1:1000 WB; 1:100 IF
Rabbit anti-PISD	Proteintech	16401-1-AP	1:500 WB
Mouse anti-Cytochrome c	Abcam	110325	1:1000 WB; 1:200 IF
Rabbit anti-BAX	Cell Signaling Technology	2772T	1:1000 WB; 1:200 IF
Rabbit anti-BAK	Cell Signaling Technology	12105T	1:1000 WB
Rabbit anti-HTRA2/Omi	Proteintech	15775-1-AP	1:1000 WB
Rabbit anti-SMAC/DIABLO	Proteintech	10434-1-AP	1:500 WB
Rabbit anti-AIF	Proteintech	67791-1-Ig	1:1000 WB
Mouse anti-ATP Synthase β	Invitrogen (Molecular Probes	A21351	1:1000 WB; 1:200 IF
Rabbit anti-Tom20	Abcam	ab78547	1:1000 WB; 1:200 IF
Mouse anti-GAPDH	Santa Cruz	sc-365062	1:2000 WB
Rabbit anti-GM130	Transduction labs	G65120	1:1000 WB
Mouse anti-OPA1	BD Biosciences	612606	1:2000 WB
Mouse anti-Tubulin	Sigma-Aldrich	T9026, mouse, clone DM1-α	1:4000 WB
Mouse anti-DLP1	BD Transduction Laboratories	611112	1:1000 WB; 1:200 IF
Rabbit anti-PARP	Cell Signaling Technology	9542T	1:1000 WB

Rabbit anti-Caspase-3	Cell Signaling Technology	9962S	1:1000 WB
Goat anti-rabbit IgG Texas Red secondary	Vector Laboratories	TI-1000	1:500 IF
Horse anti-mouse IgG fluorescein secondary	Vector Laboratories	FI-2000	1:500 IF
Goat anti-rabbit IRDye 800CW	LICOR	926-32211	1:15,000 WB
Goat anti-mouse IRDye 680RD	LICOR	926-68070	1:15,000 WB
Goat anti-mouse IgG HRP conjugate	Bio-Rad	1705047	1:2000 WB
Goat anti-rabbit IgG HRP conjugate	Bio-Rad	1706515	1:5000 WB

Supplementary Movies

Supplementary Movie 1

Time-lapse imaging of cytochrome c release in control cell upon apoptosis induction. Control siRNA-treated cell expressing cytochrome c-GFP (green) was treated at 4 min with ABT-S (10 μM ABT-737 and 2 μM S63845) in the presence of the pan-caspase inhibitor Q-VD-OPh (20 μM, 1-hour preincubation). Scale bar: 10 μm. Time interval: 1 min.

Supplementary Movie 2

Mitochondrial shape change in control cell upon apoptosis induction. Control siRNA-treated cell expressing Mito-dsRed (orange) was treated as described above. Scale bar: 10 μm. Time interval: 1 min.

Supplementary Movie 3

Cytochrome c kinetics in LACTB knockdown (KD) cell upon apoptosis induction. LACTB KD cell expressing cytochrome c-GFP (green) was treated at 4 min with Q-ABT-S as described above. Scale bar: 10 μm. Time interval: 1 min.

Supplementary Movie 4

Mitochondrial shape change in LACTB KD cell upon apoptosis induction. LACTB KD cell expressing cytochrome c-GFP (green) was treated as described above. Scale bar: 10 μm. Time interval: 1 min.

Supplementary Movie 5

Time-lapse imaging showing mitochondrial depolarization in a control siRNA-treated cell following apoptosis induction. Mitochondrial membrane potential was monitored using TMRE (orange). Cells were treated with ABT-S (10 μM ABT-737 and 2 μM S63845) at 2 min post-acquisition, in the presence of the pan-caspase inhibitor Q-VD-OPh (20 μM, pre-incubated for 1 hour). Scale bar: 10 μm. Time interval: 2 min.

Supplementary Movie 6

Time-lapse imaging showing mitochondrial depolarization in a LACTB knockdown cell treated as in Movie 5. Mitochondrial membrane potential was detected using TMRE (orange). Scale bar: 10 μm. Time interval: 2 min.

Supplementary Movie 7

Time-lapse imaging of LACTB (1 μM) catalysing fission of membrane nanotubes containing 25 mol% cardiolipin. Scale bar: 10 μm. Time interval: 0.27 sec.

Table S2.

Source data for all graphs presented in the manuscript.

Data S1 (Uncropped western blots):

Uncropped western blots and gels (Other Supplementary materials)

Fig1. A

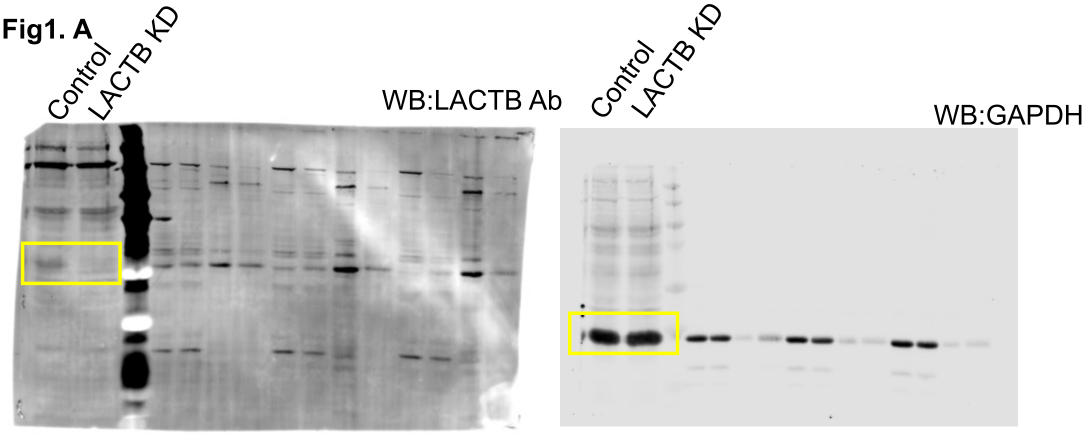

Fig1. G

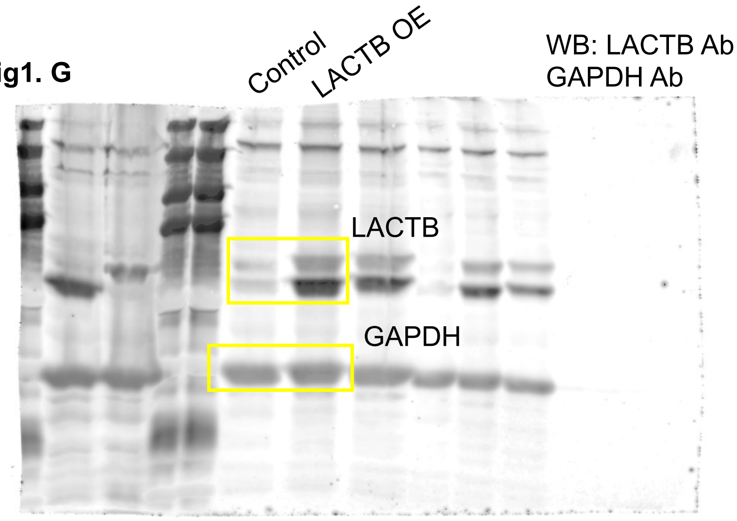

Regions enclosed in yellow boxes represent areas shown in the corresponding figures.

Uncropped western blots and gels

Fig2. A

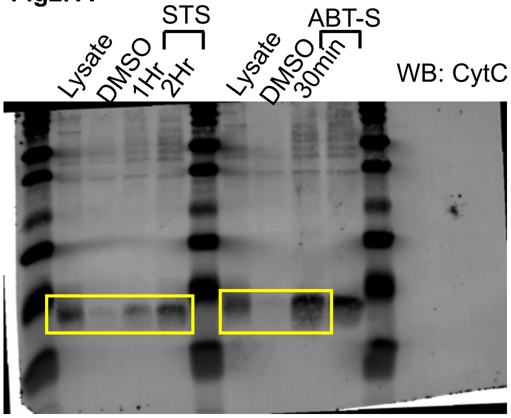

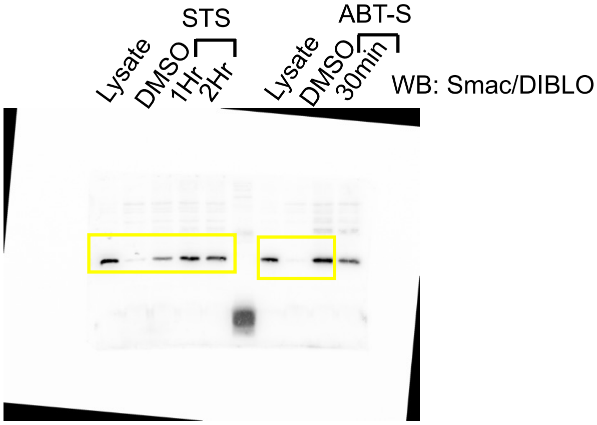

Fig2. A

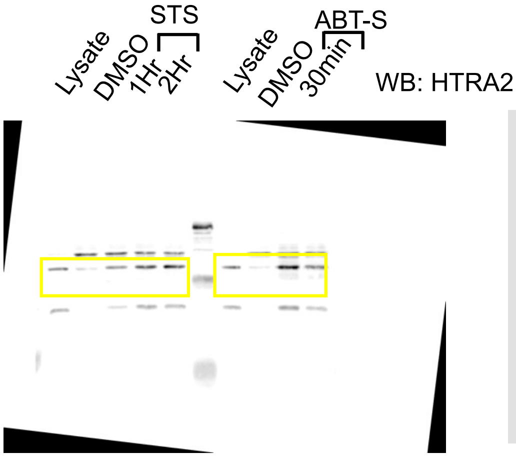

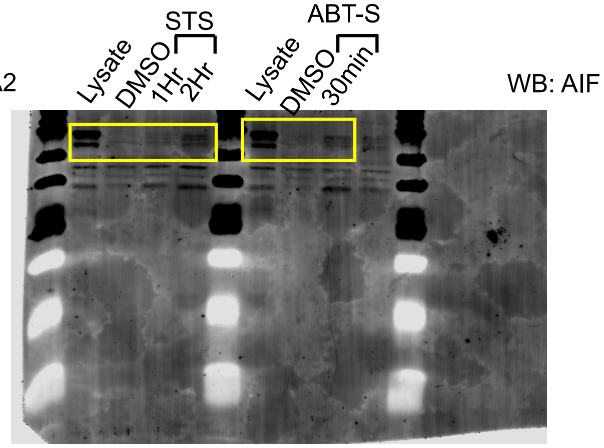

Fig2. A

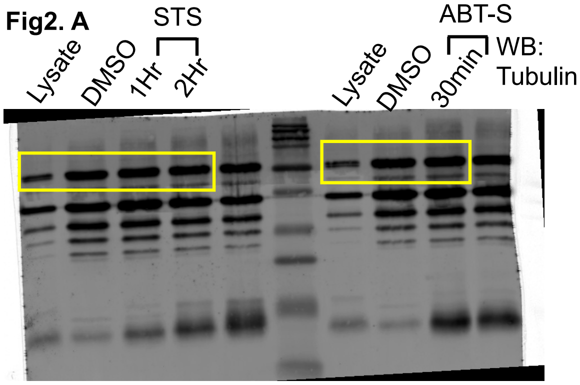

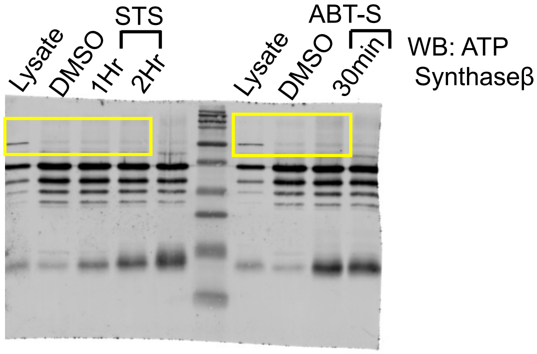

Uncropped western blots and gels

Fig2. B

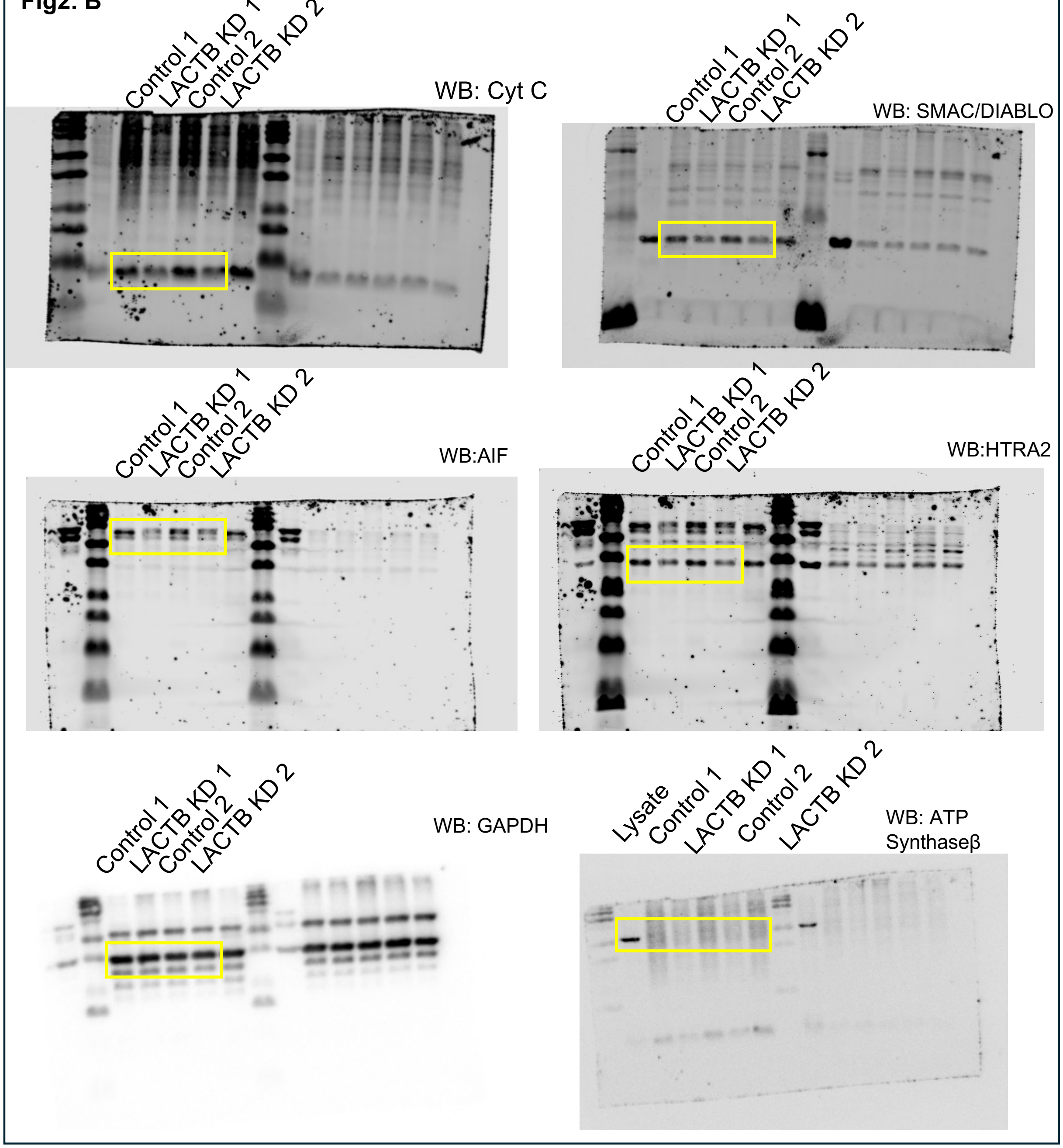

Uncropped western blots and gels

Fig2. C

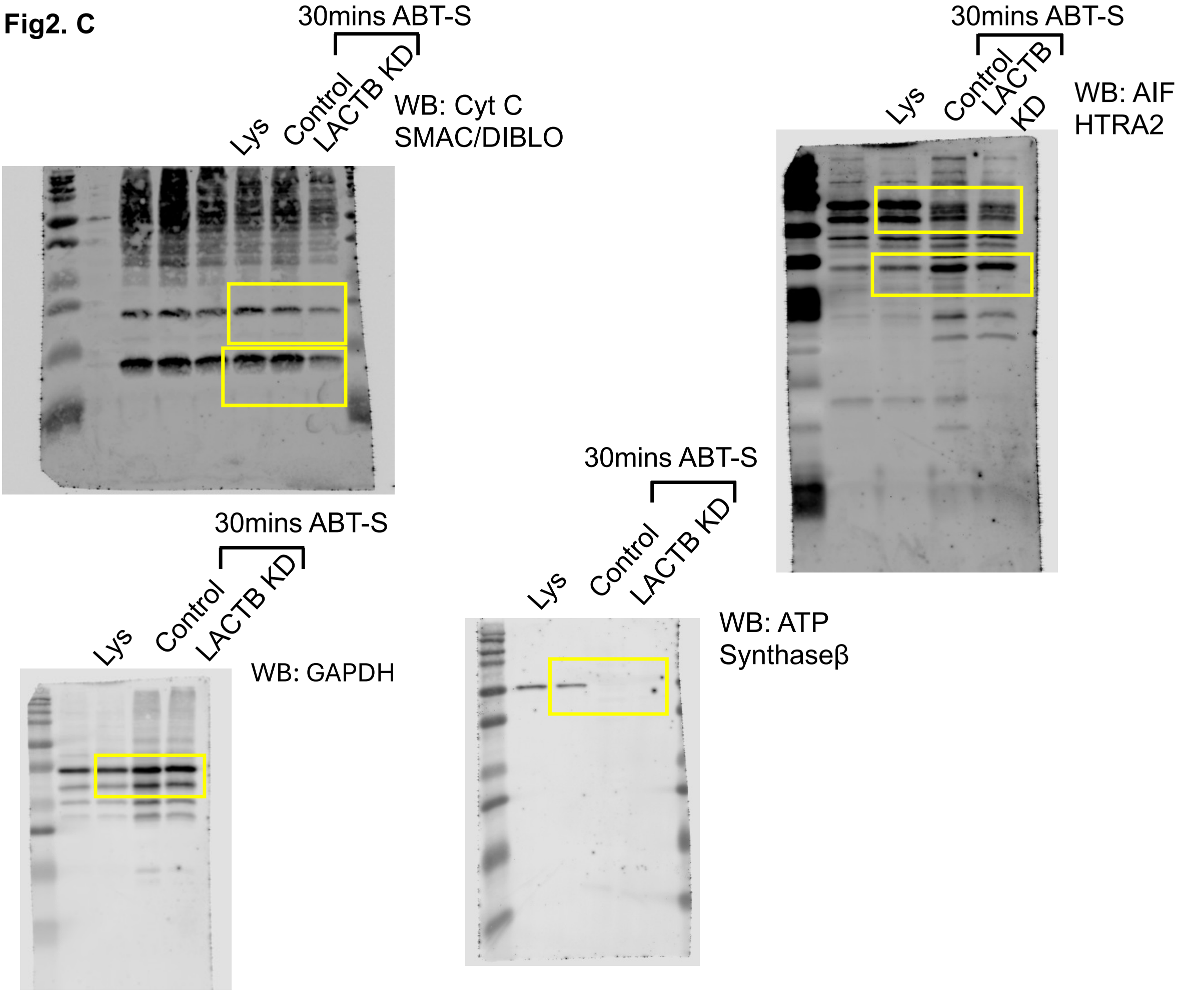

Fig2. G Cytosol with 1 hr STS

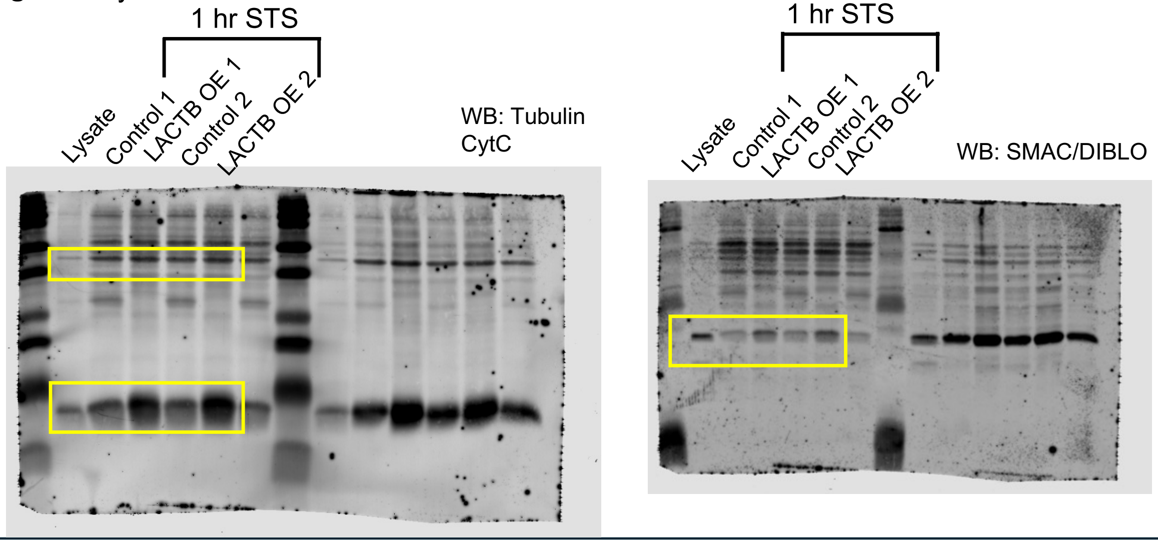

Uncropped western blots and gels

Fig2. G Cytosol with 1 hr STS

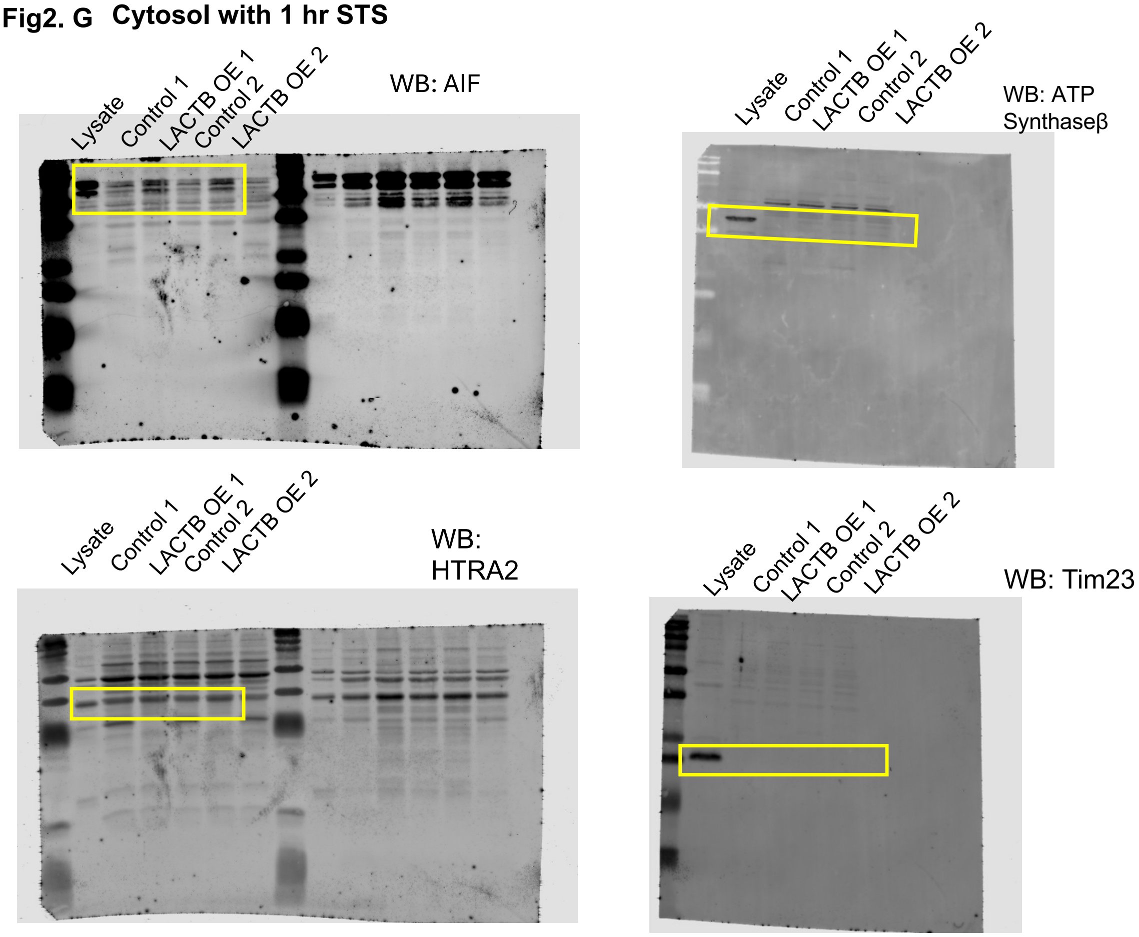

Fig2. H

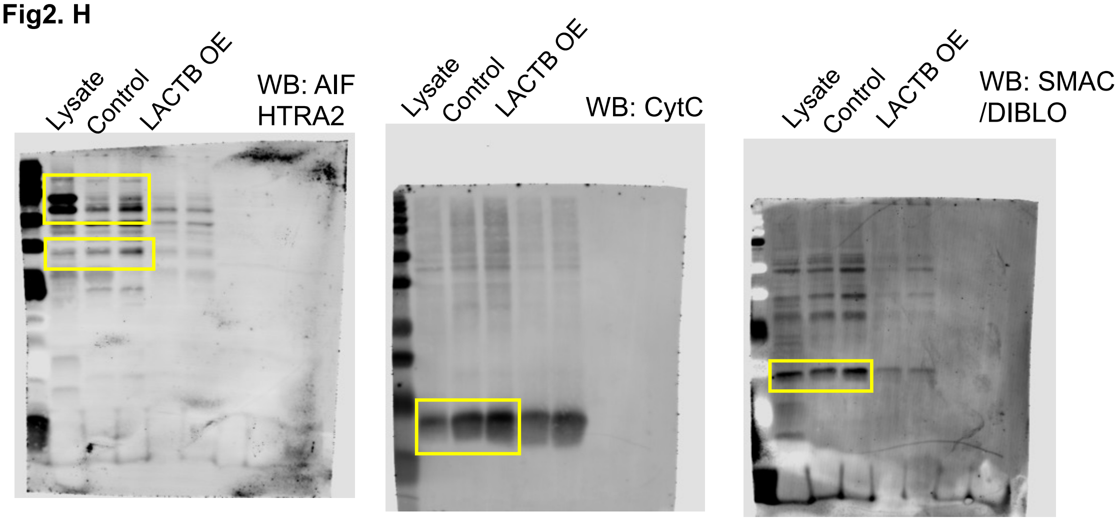

Uncropped western blots and gels

Fig2. H

Lysate Control LACTB OE

WB: ATP Synthaseβ GAPDH

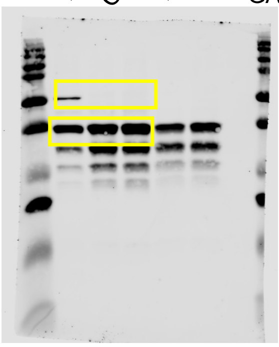

FigS1. D

Control LACTB KD

WB:LACTB Ab

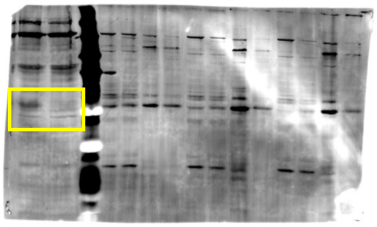

Control LACTB KD

WB:GAPDH

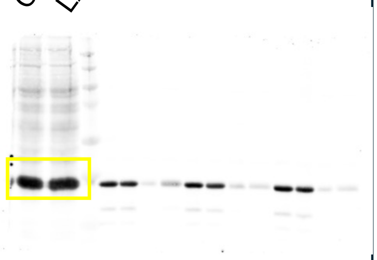

FigS2.A

FigS2.B

Control 1 LACTB KD 1 Control 2 LACTB KD 2 Control 1 LACTB OE 1 Control 2 LACTB OE 2

WB: PISD

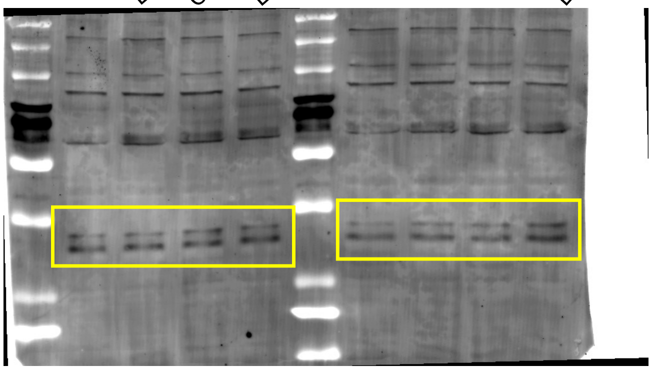

Control 1 LACTB KD 1 Control 2 LACTB KD 2 Control 1 LACTB OE 1 Control 2 LACTB OE 2

WB: GAPDH

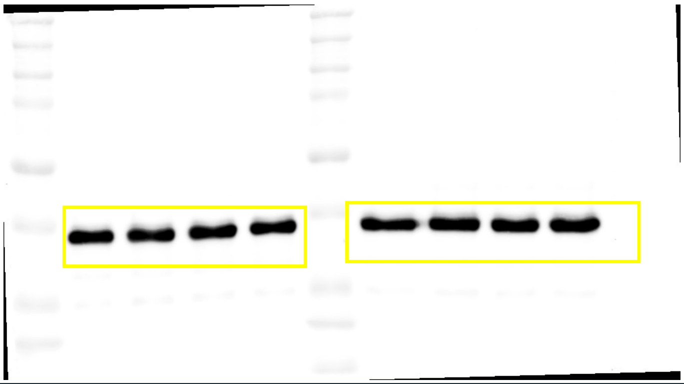

Uncropped western blots and gels

FigS2.C

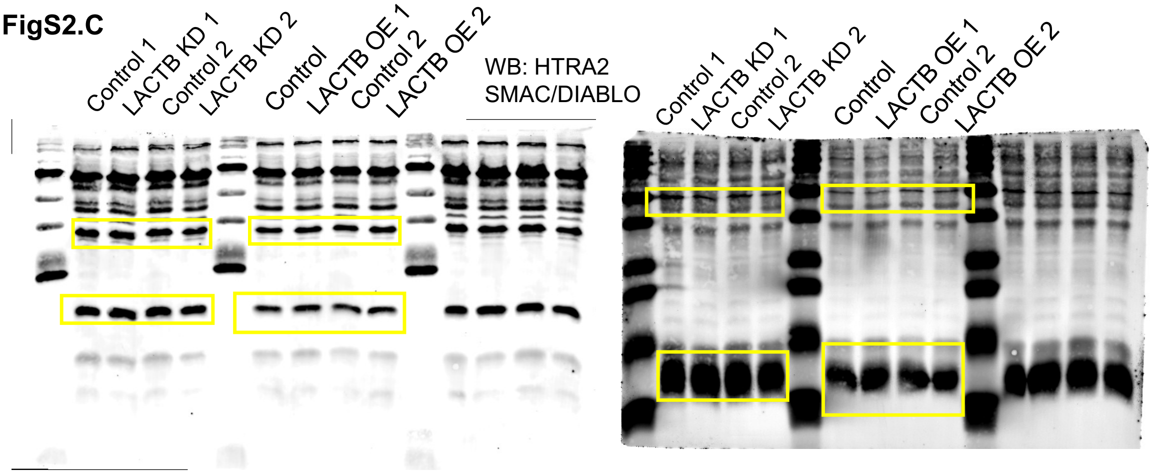

FigS2.C

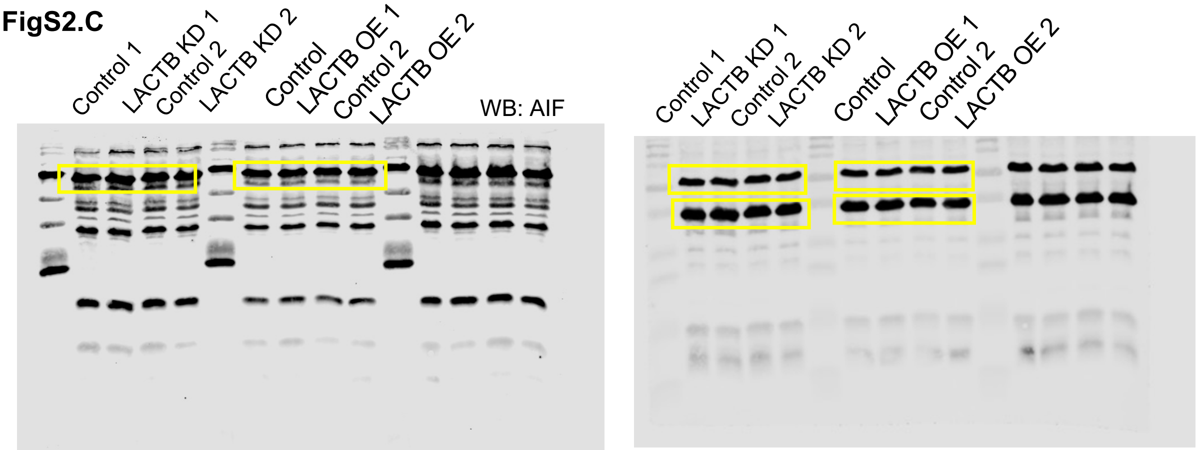

Uncropped western blots and gels

FigS3.A

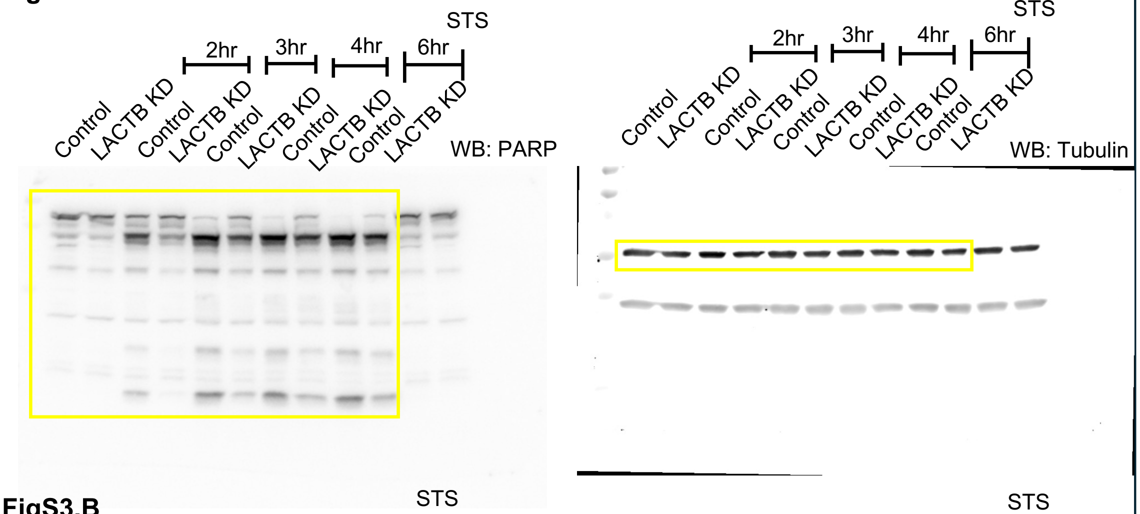

FigS3.B

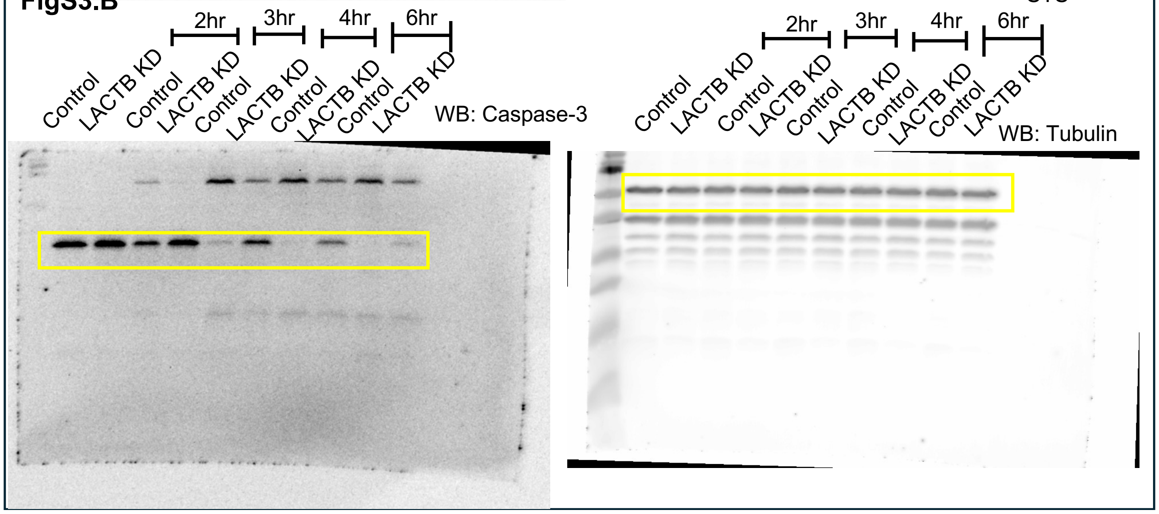

Uncropped western blots and gels

FigS4.A

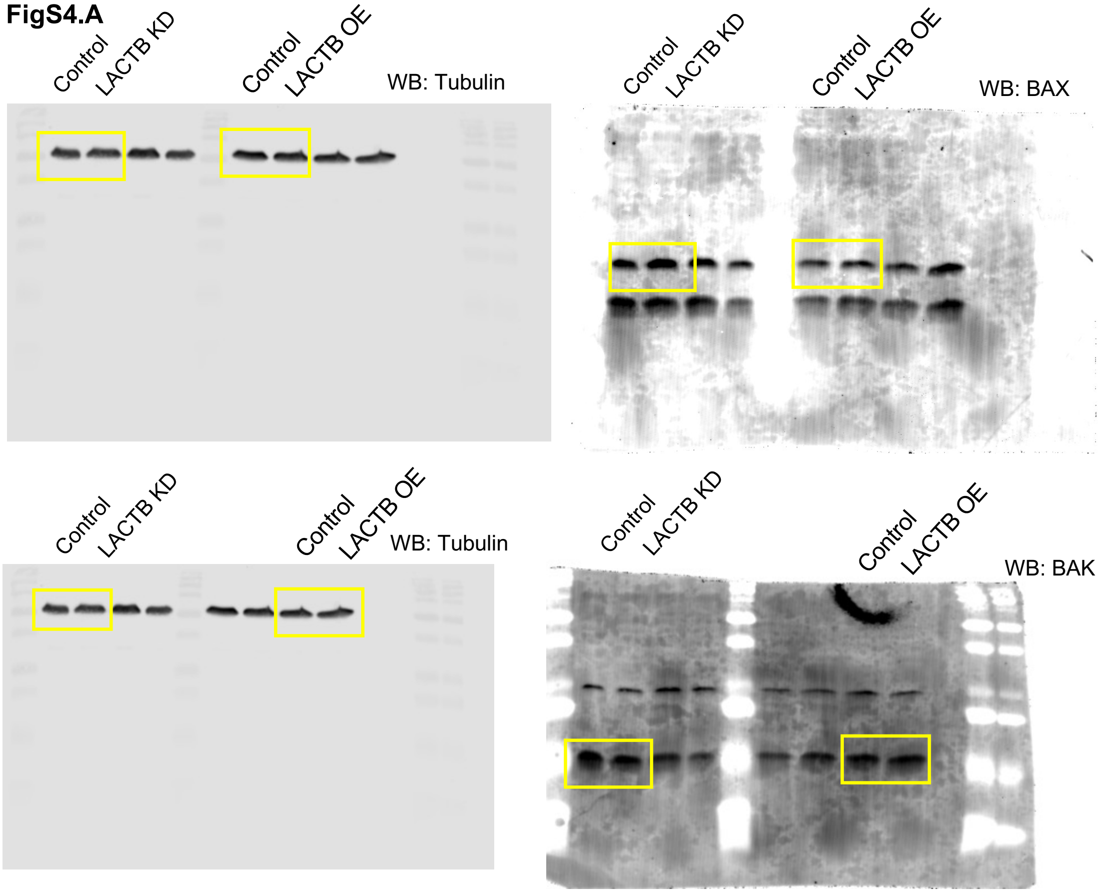

FigS4.E

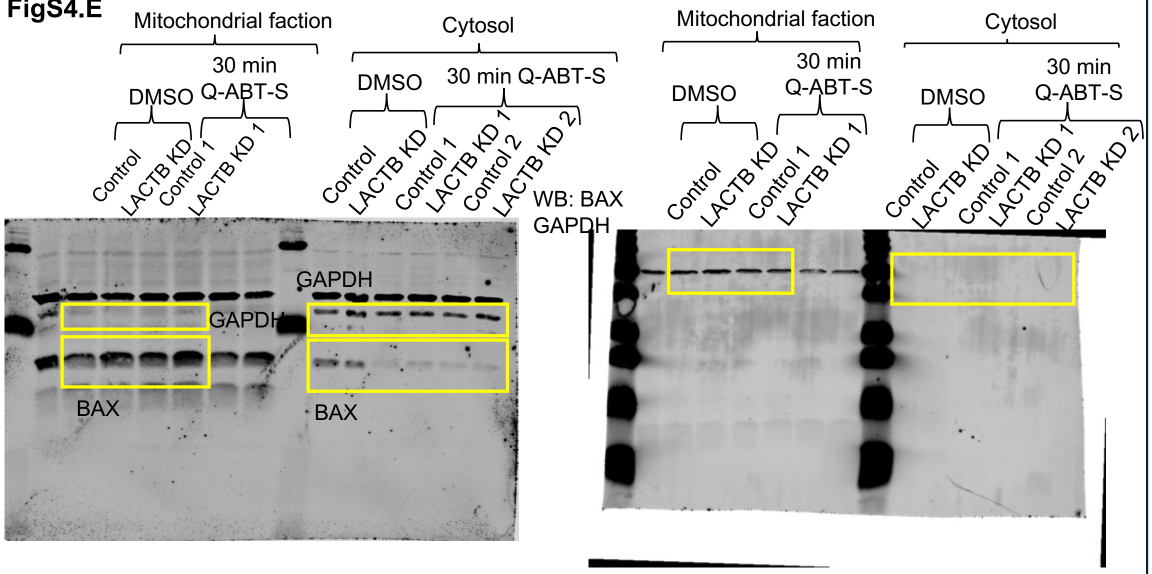

Uncropped western blots and gels

FigS10.G

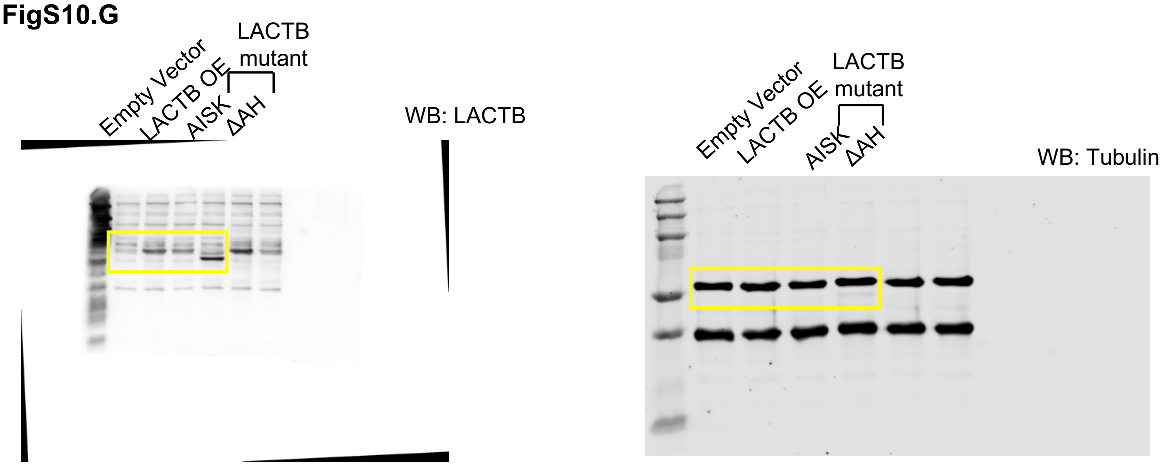

FigS10.H

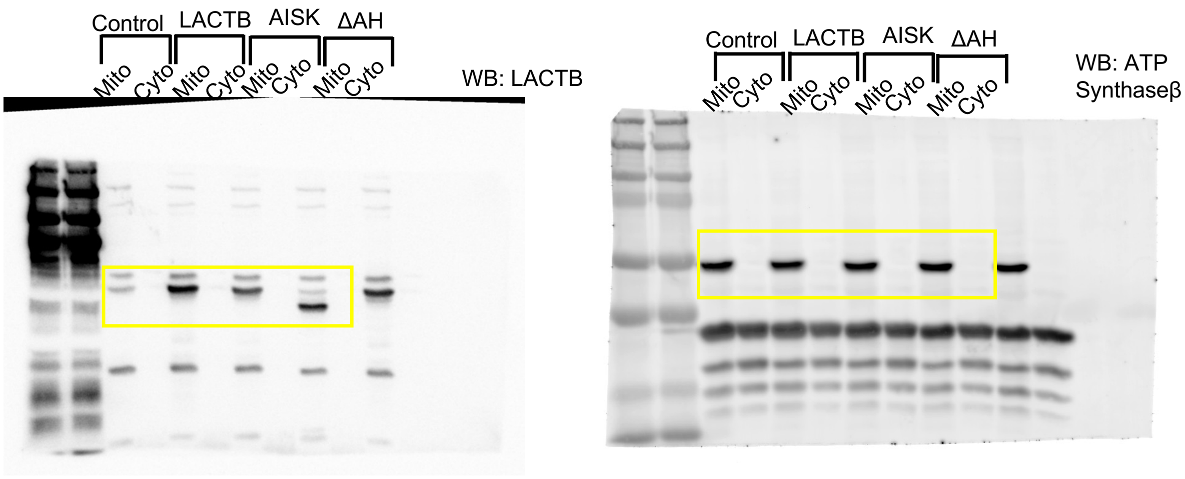

FigS11.A

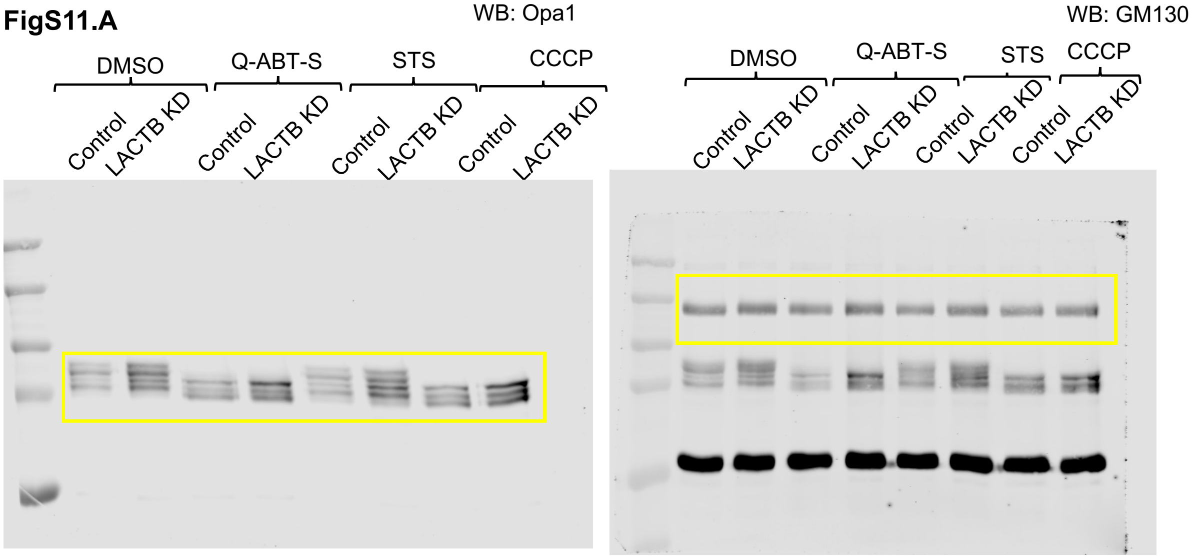

Uncropped western blots and gels

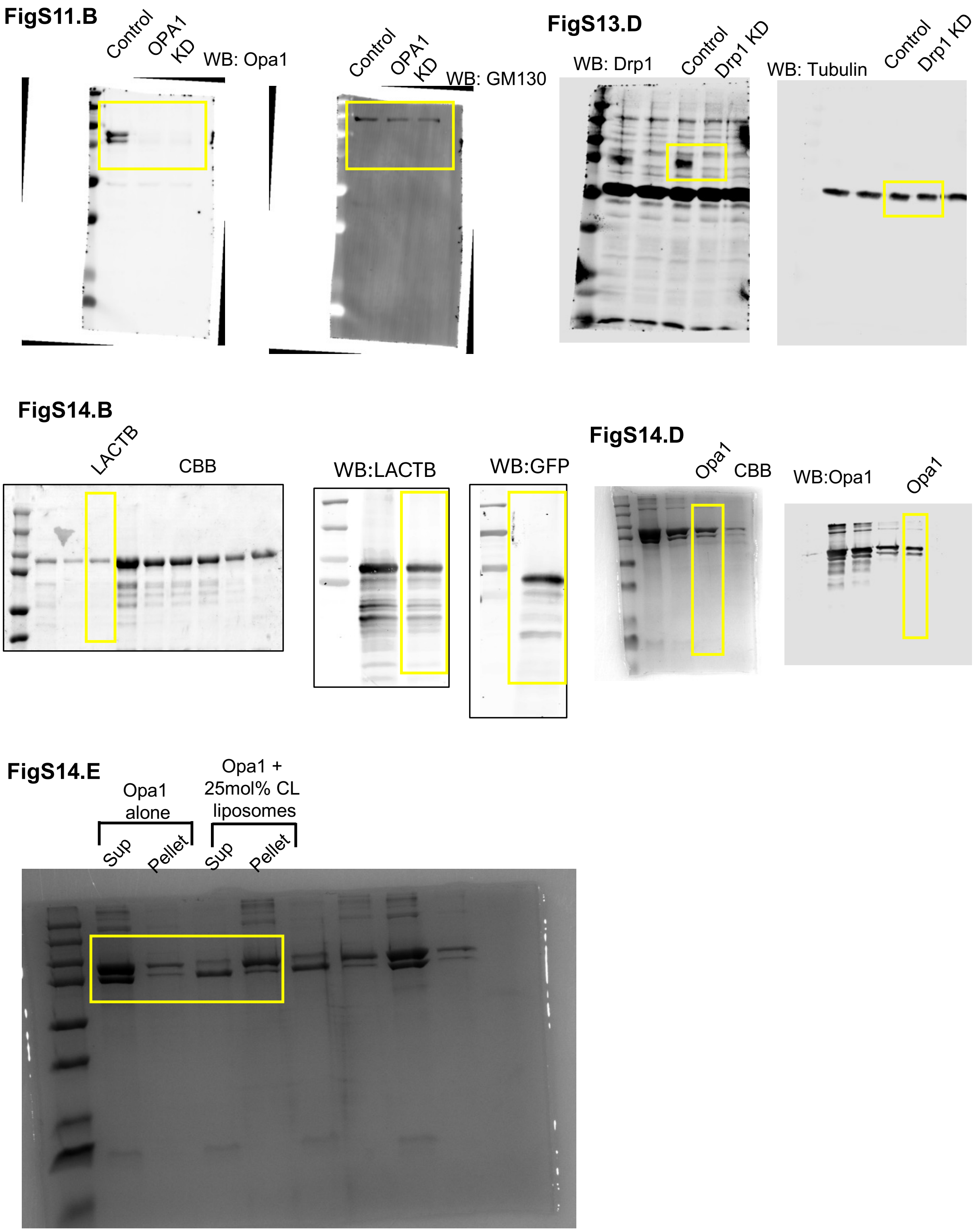

Supplement: Supplementary file 1 — Figs. S1 to S15 Table S1 Legend for table S2 Legends for movies S1 to S7 Data S1 (Uncropped Western blots) [file sciadv.adx7809_sm.pdf]
